# NF-κB in inflammation and cancer

**DOI:** 10.1038/s41423-025-01310-w

**Published:** 2025-06-25

**Authors:** Hongmei Mao, Xiaocui Zhao, Shao-cong Sun

**Affiliations:** 1 Institute for Immunology, Chinese Institutes for Medical Research, Beijing, China; 2 School of Basic Medicine, Capital Medical University, Beijing, China

**Keywords:** NF-κB, Immune response, Inflammation, Autoimmune and inflammatory diseases, Cancer, Chronic inflammation, Immunosurveillance, NF-kappaB

## Abstract

Nuclear factor-κB (NF-κB) is a family of transcription factors that transactivates genes associated with a wide range of biological processes, including immune responses, inflammation, cell growth and survival. Dysregulated NF-κB activation contributes to acute and chronic inflammatory disorders, mostly through the aberrant induction of genes encoding proinflammatory factors and metabolic disorders. Abnormal NF-κB activation also influences the development and stability of regulatory T cells, contributing to the pathogenesis of autoimmune disorders. Given the critical role of inflammation in promoting oncogenesis, the proinflammatory role of NF-κB is also linked to cancer development. In addition, aberrant NF-κB activation contributes to uncontrolled tumor cell proliferation, survival, metabolism, metastasis, tumor angiogenesis and therapy resistance. These pathological functions of NF-κB highlight its potential as a therapeutic target for both inflammatory diseases and cancer. In this review, we summarize recent findings regarding the role of NF-κB in these pathological processes and discuss the underlying mechanisms. We also explore potential therapeutic strategies aimed at targeting the NF-κB pathway for disease treatment, along with an analysis of possible challenges.

## Introduction

**Fig. 1:**
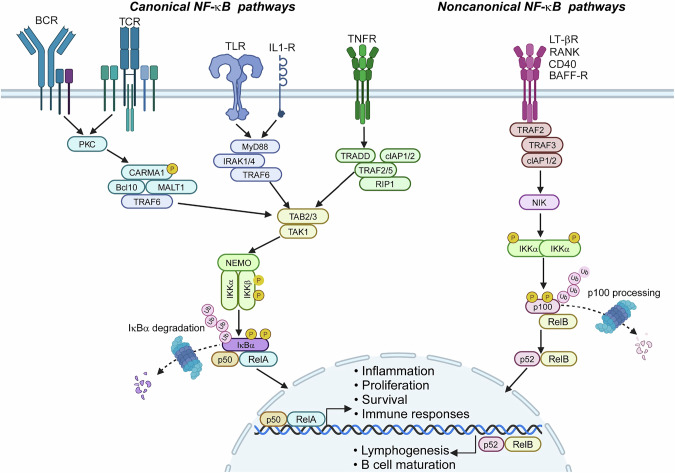
Canonical and noncanonical NF-κB signaling pathways. Canonical NF-κB signaling is activated by a variety of immune receptors, including B-cell receptors (BCRs), T-cell receptors (TCRs), Toll-like receptors (TLRs), interleukin-1 receptor (IL-1R), and tumor necrosis factor receptor (TNFR). These receptors initiate signaling cascades (e.g., CARMA1/BCL10/MALT1 or TAK1/TAB complexes) that converge on the IKK complex (IKKα/IKKβ/NEMO). Phosphorylation of IκBα by IKKβ triggers its ubiquitination and proteasomal degradation, liberating the p50/RelA dimer for nuclear translocation. This pathway drives survival, inflammation, and immunoresponsive gene expression. Noncanonical NF-κB signaling is triggered primarily by ligand engagement of TNFR superfamily members (e.g., CD40, RANK, LT-βR, and BAFF-R), which stabilize NF-κB-inducing kinase (NIK) by disrupting the c-IAP/TRAF2/3 E3 ubiquitin ligase complex. NIK activates IKKα, which phosphorylates the NF-κB2 precursor p100 to trigger its conversion to p52 via proteasomal processing. The p52/RelB dimer translocates to the nucleus, where it regulates genes critical for lymphoid organogenesis, B lymphocyte survival and maturation. The images were created with BioRender (www.biorender.com)

Nuclear factor kappa B (NF-κB) represents a family of structurally related transcription factors, including RelA (also called p65), RelB, c-Rel, NF-κB1 (p50), and NF-κB2 (p52), which exist as homo or heterodimmers. NF-κB proteins share a conserved domain known as the Rel-homology domain, which enables their dimerization, nuclear localization, DNA binding, and interaction with the inhibitory protein IkB [1–4]. Through association with IκBs, NF-κBs are sequestered in the cytoplasm as inactive complexes, and the activation of NF-κB complexes can be induced by various immune stimuli through canonical and noncanonical pathways (Fig. 1). The canonical pathway is rapidly triggered by proinflammatory stimuli, such as the cytokines TNF-α and IL-1β and the bacterial component lipopolysaccharide (LPS), as well as by antigens, which stimulate a cascade of receptor-proximal signaling events leading to the activation of an IκB kinase (IKK) complex composed of IKKα, IKKβ, and NF-κB essential modulator (NEMO, also called IKKγ) [5]. The activated IKK complex then phosphorylates IκB proteins, predominantly IκBα, resulting in their ubiquitin-dependent degradation by the proteasome, allowing the released NF-κB dimers (typically p50/RelA) to translocate to the nucleus for target gene transactivation [6]. Activation of the noncanonical NF-κB pathway is mediated mainly by members of the TNF receptor (TNFR) superfamily, such as CD40, B-cell activating factor receptor (BAFF-R), lymphotoxin-β receptor (LTβR), and receptor activator of NF-κB (RANK) [7]. Upon engagement by specific ligands, these TNFRs transduce signals that target the disruption of an E3 ubiquitin ligase complex composed of TRAF2, TRAF3, and the cellular inhibitor of apoptosis protein 1 (c-IAP1) or c-IAP2. Under stable conditions, this c-IAP/TRAF E3 complex mediates ubiquitin-dependent degradation of the noncanonical NF-κB-inducing kinase (NIK) to prevent its signaling function [8]. TNFR-induced disruption of this E3 complex results in stabilization of NIK, allowing NIK to phosphorylate and activate its downstream kinase, IKKα. Activated IKKα then phosphorylates p100, the NF-κB2 precursor protein containing both p52 and a C-terminal IκB-like structure capable of inhibiting NF-κB members, especially RelB [8]. The phosphorylation of p100 triggers ubiquitin-dependent degradation of its C-terminal IκB-like portion, a process (known as p100 processing) that leads to the generation of mature NF-κB2 p52 and the nuclear translocation of p52 and RelB, causing transactivation of specific target genes [9, 10] (Fig. 1). This pathway governs specialized processes such as lymphoid organ development, B-cell survival, and T-cell effector function [1].

Despite their significant differences in signaling mechanisms and functions, the canonical and noncanonical NF-κB pathways both play critical roles in the transcriptional regulation of genes vital for immune and inflammatory responses, cell survival and proliferation [11]. NF-κB promotes inflammation by driving the expression of proinflammatory cytokines (e.g., TNF-α, IL-1, and IL-6), chemokines, and cell adhesion molecules [12, 13]. NF-κB also regulates the homeostasis, activation, differentiation and effector function of regulatory T (Treg) cells [14, 15]. Hence, dysregulated NF-κB activation is associated with acute and chronic inflammatory disorders as well as autoimmune diseases [13]. Another well-documented function of NF-κB is to promote cell survival, which involves the induction of a number of apoptosis inhibitors, such as Bcl-2, Bcl-XL, c-IAP1, c-IAP2, and c-FLIP [16–21]. NF-κB also stimulates cell proliferation through the transcriptional induction of genes involved in cell cycle progression, such as Cyclin D1 [22, 23]. Aberrant NF-κB activation contributes to the uncontrolled growth and survival of malignant cells [24]. By integrating inflammatory and survival signals, NF-κB plays a central role in inflammation and inflammation-driven tumorigenesis, making it a potential therapeutic target in the treatment of inflammatory diseases and cancer. However, NF-κB-based therapies face challenges and complications due to the requirement of NF-κB for normal cell survival and immune functions. An in-depth understanding of its context-dependent mechanisms offers potential promising strategies for precision therapies.

## NF-κB in inflammation

As mentioned above, NF-κB plays a pivotal role in mediating immune and inflammatory responses. During an acute inflammatory response, NF-κB is rapidly activated by various stimuli, including pathogen-associated molecular patterns (PAMPs), damage-associated molecular patterns (DAMPs) and cytokines (e.g., TNF-α and IL-1β) [25]. Activated NF-κB in turn drives the expression of proinflammatory factors, including cytokines (e.g., IL-1, IL-6, IL-12, and TNF-α), chemokines, cell adhesion molecules, and enzymes such as inducible nitric oxide synthase (iNOS) and cyclooxygenase-2 (COX-2) [26, 27]. These factors participate in an inflammatory process that mediates the recruitment of immune cells and factors to the site of infection for pathogen elimination and tissue repair initiation [25], highlighting the critical role of NF-κB in host defense. However, persistent activation of NF-κB, which is induced by prolonged infections, autoimmune triggers, oxidative and metabolic stress (e.g., obesity), or environmental factors, results in sustained production of inflammatory factors, leading to chronic inflammation [28]. Uncontrolled NF-κB activation also promotes the activation, survival and differentiation of inflammatory T cells, such as Th17 cells, and renders self-reacting T cells resistant to suppression by Treg cells, thereby contributing to autoimmunity [15, 29]. Prolonged NF-κB signaling contributes to the pathogenesis of a variety of inflammatory and autoimmune diseases, such as rheumatoid arthritis (RA) [30], inflammatory bowel disease (IBD) [31, 32], systemic lupus erythematosus (SLE) [33], atherosclerosis [34], neurodegenerative diseases [35], and chronic obstructive pulmonary disease (COPD) [36]. Thus, NF-κB acts as a double-edged sword: it is indispensable for host defense during acute inflammation but detrimental when it is chronically activated. A better understanding of its mechanisms of action is important for developing new therapeutic strategies for treating inflammatory diseases.

### Role of NF-κB in immune and inflammatory responses

Acute inflammation is an immediate and short-term response of the immune system to harmful stimuli, such as pathogens and tissue injury. It serves as the first line of protection, aiming at rapidly eliminating or controlling infections and tissue damage, clearing damaged cells, and initiating tissue repair, thereby maintaining homeostasis and preventing pathogen spread [37, 38]. The classic signs of acute inflammation include redness, heat, swelling, pain, and loss of function, which are caused by increased blood flow, increased vascular permeability, the release of pain-inducing mediators and pressure from swelling, and tissue damage [39].

**Fig. 2:**
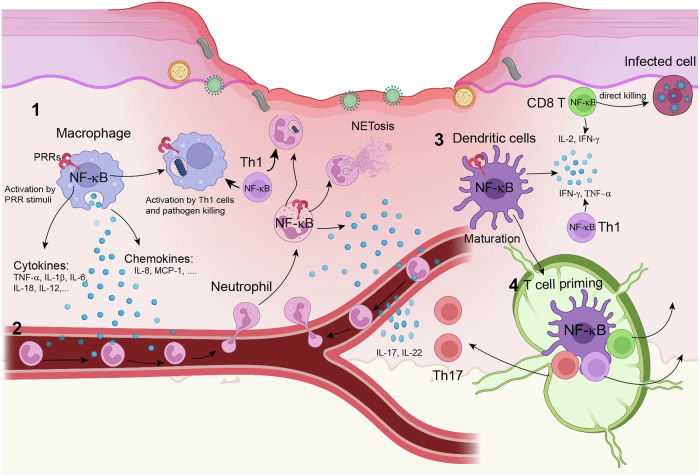
NF-κB in acute inflammation. During an acute inflammatory response, NF-κB signaling orchestrates a coordinated immune response involving macrophages, neutrophils, dendritic cells (DCs), and T cells. 1. Tissue-resident macrophages sense infections and tissue injury through PRR-mediated recognition of PAMPs and DAMPs, leading to the activation of NF-κB and the induction of proinflammatory cytokines and chemokines. 2. Macrophage-derived cytokines and chemokines recruit leukocytes, particularly neutrophils, to inflamed tissue, where they are activated via PRRs and cytokine receptors, triggering NF-κB signaling and the production of proinflammatory cytokines to increase inflammation and phagocytosis. 3. DCs in inflamed tissue capture pathogen-derived antigens and are stimulated for maturation and proinflammatory cytokine production in an NF-κB-dependent manner. 4. Mature DCs migrate to lymph nodes, where they present antigens to T cells to trigger their activation and differentiation. Effector T cells migrate to inflamed tissue, where Th1 cells amplify inflammation and promote pathogen killing by activating neutrophils and macrophages, and CD8 T cells mediate the lysis of infected cells. The images were created with BioRender (www.biorender.com)

Acute inflammation is a coordinated, multistep response that includes recognition of injury or infection, vascular changes, leukocyte recruitment and activation, phagocytosis and pathogen clearance, and resolution and repair [38, 40]. This process is initiated through the detection of PAMPs and DAMPs by pattern recognition receptors (PRRs) on innate immune cells, particularly tissue macrophages, which trigger the production of inflammatory cytokines (e.g., TNF, IL-1, and IL-6) and chemokines (e.g., CCL2 and CXCL8) [41–45] (Fig. 2). These mediators then act on local blood vessels to induce vasodilation, increase vascular permeability and the expression of endothelial cell adhesion molecules, such as selectins and intercellular adhesion molecule 1 (ICAM-1), causing the release of fluid and proteins and the migration of leukocytes from the bloodstream to infected tissue [37, 46]. Following extravasation, leukocytes migrate to the site of infection or injury in the direction of chemokines. Neutrophils are the primary early responders in an inflammatory response, and they attack pathogens via a number of mechanisms, including phagocytosis, granule secretion, and the formation of extracellular traps [47]. Although less prominent in the early phase, macrophages and dendritic cells (DCs) are subsequently recruited to the affected tissue and contribute to pathogen clearance and tissue repair [48, 49]. Pathogen destruction and tissue repair also involve the interplay between neutrophils and macrophages [50]. In addition, tissue mast cells, which are better known for their role in allergic reactions, also participate in the modulation of inflammatory responses, and they act by releasing preformed mediators (e.g., histamine, antimicrobial peptides) and cytokines to amplify or suppress the inflammatory process [51]. The inflammatory response is typically self-limiting, as the triggers are resolved, and this spontaneous decline is driven by intrinsic regulatory mechanisms. First, short-lived mediators (e.g., histamine and prostaglandins) are rapidly degraded after release. Second, neutrophils undergo programmed apoptosis within hours, preventing prolonged tissue damage. Third, active termination pathways shift the balance from proinflammatory signals (e.g., TNF-α and IL-1β) to anti-inflammatory mediators, such as lipoxins, TGF-β, and IL-10, which suppress inflammation and promote repair [52–54].

Unlike neutrophils and macrophages, T cells are not frontline responders to an infection, but they play a crucial role in regulating the process of inflammation and pathogen clearance (Fig. 2). The delayed response of T cells is due to the time-consuming process of their activation, clonal expansion and differentiation before their recruitment to infected tissue [55, 56]. This process, which takes several days, is initiated when naïve T cells encounter pathogen-derived antigens such as peptide‒MHC complexes on DCs in peripheral lymphoid organs. In the presence of different cytokines secreted by DCs or macrophages, CD4^+^ T cells differentiate into distinct subsets of effector T helper (Th) cells with specialized functions [57]. For example, Th1 cells activate macrophages to destroy phagocytosed pathogens via the production of the macrophage-activating cytokine IFN-γ and the expression of cell surface molecules, such as the CD40 ligand. Th17 cells secrete IL-17 and related cytokines that promote neutrophil recruitment to increase extracellular pathogen destruction and inflammation [58–60]. On the other hand, Treg cells can produce IL-10 and TGF-β to suppress excessive inflammation and prevent tissue damage [61]. In addition, antigen-specific CD8^+^ T cells differentiate into cytotoxic T lymphocytes (CTLs), which kill infected cells and thus limit inflammation [62].

While acute inflammation is a vital and tightly regulated defense mechanism that protects the body from injury and infection, excessive or unresolved inflammation can drive severe pathological conditions, underscoring the need to elucidate its molecular mechanisms. A deeper understanding of these mechanisms offers promising therapeutic avenues for inflammatory disorders. As highlighted earlier, the transcription factor NF-κB serves as a central orchestrator of acute inflammation (Fig. 2). Below, we discuss how NF-κB regulates key innate immune cells, neutrophils, macrophages, DCs, and T lymphocytes to coordinate inflammatory responses, pathogen clearance, and resolution.

#### NF-κB in macrophage regulation

Tissue macrophages are crucial for initiating an acute inflammatory response to infections and tissue injury (Fig. 2). They express a large variety of PRRs, including toll-like receptors (TLRs), nucleotide-binding oligomerization domain-like receptors (NLRs), C-type lectin receptors, RIG-I-like receptors (RLRs), and cytosolic DNA sensors, which sense invading microorganisms through recognition of PAMPs and tissue injury through recognition of DAMPs [41, 63]. Upon stimulation, PRRs transduce signals that activate macrophages to secrete inflammatory mediators, including proinflammatory cytokines, such as TNF-α, IL-1β, and IL-6; chemokines, such as CXCL8 (also called IL-8) and CCL2 (also called MCP-1); and lipid mediators, which in turn mediate leukocyte recruitment to the site of infection or tissue injury. While different PRRs signal through distinct adapter molecules, they all target the activation of IKK and canonical NF-κB [64]. Activated NF-κB directly engages in the transactivation of genes encoding proinflammatory cytokines, and it also promotes the generation of lipid inflammatory mediators, including prostaglandins and leukotrienes, through the transcriptional induction of the enzyme cyclooxygenase (COX-2). NF-κB also regulates macrophage functions through the induction of immunoregulatory cytokines, such as IL-12 and IL-23 [65, 66]. Genetic evidence suggests an essential role for the NF-κB member c-Rel in mediating the induction of *Il12b*, the gene encoding the p40 subunit of IL-12 and IL-23 [65]. c-Rel binds to the *Il12b* promoter to activate its transcription, and c-Rel deficiency in macrophages severely reduces the expression of IL-12 p40 at the mRNA and protein levels [65]. c-Rel, as well as RelA, are also critically involved in TLR4-induced expression of the *Il12a* and *Il23a* genes, which encode IL-12 p35 and IL-23 p19, respectively [66]. IL-12 and IL-23 promote immune and inflammatory responses via diverse mechanisms, including the induction of inflammatory Th1 and Th17 cells and the activation of macrophages and natural killer (NK) cells [67, 68]. Another NF-κB member, p50, is also involved in macrophage activation. Mice deficient in both c-Rel and p50 exhibit compromised innate immunity against bacterial sepsis, with macrophages demonstrating impaired phagocytic activity, diminished bacterial clearance, and reduced production of antimicrobial peptides [69].

The negative regulation of NF-κB in macrophages contributes to the resolution of inflammation. For example, macrophage-derived IL-10 inhibits the activation of NF-κB to suppress proinflammatory cytokine gene transcription, thus preventing excessive inflammation [70]. Additionally, the deubiquitinating enzyme A20 inhibits the canonical NF-κB pathway in macrophages to control inflammation during the resolution phase [71].

#### NF-κB in neutrophil recruitment and functional regulation

One important function of the proinflammatory mediators produced by tissue macrophages is to induce leukocyte recruitment to the site of infection or tissue injury, which is accomplished mainly by endothelial activation (Fig. 2). TNF-α and IL-1β activate the canonical NF-κB pathway in endothelial cells (ECs), thereby stimulating the production of cell adhesion molecules, such as E-selectin and ICAM-1, which facilitate neutrophil transmigration to sites of infection or injury [72]. Activated NF-κB also enhances epithelial cell expression of IL-8/CXCL8, a key chemokine that induces chemotaxis of neutrophils and other granulocytes, directing their migration toward inflamed tissues [73]. Like tissue macrophages, neutrophils express PRRs that detect PAMPs and DAMPs to trigger the activation of NF-κB and the induction of genes encoding proinflammatory cytokines and chemokines for endothelial cell activation [74]. In addition, NF-κB-induced COX-2 generates prostaglandins, which promote vasodilation and influence neutrophil migration dynamics [75]. Thus, NF-κB activation in neutrophils plays an important role in mediating amplification of the inflammatory response [76–78].

NF-κB also regulates the function and survival of neutrophils. NF-κB-driven iNOS produces NO, modulating neutrophil functions such as chemotaxis, adhesion, phagocytosis, respiratory burst, and apoptosis [79]. Importantly, the role of NF-κB in regulating neutrophil function and inflammation is complex. Attenuated NF-κB activation has been shown to induce neutrophil apoptosis [80]; however, complete inactivation of NF-κB promotes neutrophil survival, and this surprising action involves sustained activation of the MAP kinase p38 and elevated expression of the apoptosis inhibitor Bcl-xL [36]. Moreover, compelling evidence suggests that prolonged inhibition of the canonical NF-κB pathway in mice by myeloid cell-conditional IKKβ ablation or treatment with IKKβ inhibitors promotes IL-1β secretion and acute inflammation induced by the endotoxin LPS [43]. Specifically, the inhibition of IKKβ sensitizes mice to LPS-induced septic shock, which is characterized by increased serum levels of IL-1β, despite the reduction in TNF-α (43). In neutrophils, NF-κB inhibits the processing of pro-IL-1β to mature IL-1β by suppressing the activity of pro-IL-1β-processing proteases [43]. These results are corroborated by a recent finding that a patient with an inherited NFKBIA mutation, generating a dominant-negative IκBα mutation that severely represses NF-κB activation, displays neutrophil-mediated autoinflammation as a result of aberrant IL-1β secretion [81]. Impaired NF-κB activation is also associated with increased granulocytosis and neutrophilia [43, 81]. Together, these findings raise concerns about the serious side effects of long-term NF-κB inhibition. However, since partial or transient IKKβ inhibition does not lead to IL-1β abnormalities [43], targeting NF-κB is still a viable approach for the treatment of inflammatory diseases. Moreover, the anti-inflammatory function of IKKβ/NF-κB appears to be specific for some, but not all, inflammation models. For example, in a mouse model of traumatic spinal cord injury (SCI), myeloid cell-specific IKKβ deletion significantly reduces infiltration of neutrophils, as well as macrophages, coupled with ameliorated neuroinflammation and neuronal damage, thus contradicting the results obtained with the LPS-induced septic shock model [82]. Clearly, a better understanding of the role of NF-κB in different inflammatory conditions is crucial for therapeutic approaches.

#### NF-κB in DC regulation

DCs are a heterogeneous population of antigen-presenting cells (APCs) that bridge innate and adaptive immunity [83]. They express the major histocompatibility complex class I (MHC I) and MHC II molecules required for the presentation of antigen peptides to the T-cell receptor (TCR) on CD8 and CD4 T cells, respectively. DCs normally exist in an immature state and present self-peptides to T cells to induce immune tolerance, an immunoregulatory mechanism that maintains immune homeostasis and prevents autoimmunity [84]. During acute inflammation, DCs are stimulated by PAMPs via their PRRs and undergo maturation, including the upregulation of MHC I and MHC II and the induction of costimulatory molecules, such as CD80 (or B7-1), CD86 (or B7-2), and CD40, to prepare them for efficient antigen presentation and T-cell activation. Upon stimulation, DCs also start expressing the chemokine receptor CCR7, guiding them to migrate to regional lymph nodes, where they encounter and activate naïve CD4^+^ and CD8^+^ T cells to initiate an adaptive immune response. The role of NF-κB in DCs is multifaceted and includes development, maturation, survival and cytokine production [85] (Fig. 2). A well-known function of NF-κB is to mediate DC maturation. Stimuli of DC maturation are typical inducers of the canonical NF-κB pathway, and blocking NF-κB activation with pharmacological inhibitors or via adenoviral transfer of IκBα inhibits DC maturation by preventing the expression of MHC-II and costimulatory molecules [86, 87]. Among the NF-κB family members, RelB appears to be critical for DC maturation, since genetic ablation or siRNA-mediated knockdown of RelB inhibits the maturation and T-cell-priming function of DCs [88, 89]. Consistently, DCs deficient in the NF-κB2 precursor protein p100, the primary inhibitor of RelB, display increased RelB activation, which is associated with increased expression of MHC II and costimulatory molecules as well as increased CD4^+^ T-cell activation ability [90]. RelB can be activated via both canonical and noncanonical NF-κB signaling pathways [1]. In DCs stimulated with TLR ligands, RelB is activated by the canonical NF-κB pathway, as a RelB/p50 heterodimer is required for the induction of DC maturation and cytokine production [91]. Consistently, genetic evidence suggests that the noncanonical NF-κB pathway is dispensable for DC development and maturation but plays an important role in mediating antigen cross-presentation and the induction of IL-12 p40, a common subunit of the proinflammatory cytokines IL-12 and IL-23 [92–94]. Another NF-κB family member, c-Rel, also participates in DC maturation, but it appears to act indirectly via the induction of RelB expression [91]. The role of c-Rel in DC regulation may be more complex and beyond maturation, since c-Rel-deficient bone marrow-derived DCs are compromised in T-cell activation despite their normal maturation after LPS treatment [95]. The exact mechanism by which c-Rel regulates DC function remains to be investigated, but c-Rel also mediates the TLR-stimulated expression of cytokines, including IL-12 p70.

NF-κB is also involved in the development of DCs. Initial studies demonstrated an essential role for RelB in the development of the CD11c^+^CD8a^–^ DC subset in mice. RelB has also been shown to mediate the development of the human monocyte-derived DC subset [96]. Subsequent studies revealed the involvement of p50 and RelA in DC development [85]. Mutant mice deficient in both p50 and RelA are largely defective in generating CD11c^+^ DCs. Although double deficiency in p50 and c-Rel does not affect DC development, it impairs DC survival and IL-12 production induced by CD40L and RANKL [85].

#### Role of NF-κB in T cells

NF-κB is critical for different aspects of T-cell functions, including development, activation, differentiation, effector function, and memory responses. During the development of T cells in the thymus, NF-κB activation by the pre-TCR in CD4^–^CD8^–^ double-negative (DN) thymocytes is important for their survival and progression to the double-positive (DP) stage [97], although this function is somewhat controversial [98, 99]. Another function of NF-κB is to regulate thymocyte selection, a mechanism that generates functional single-positive (SP) thymocytes [100]. NF-κB inhibition by transgenic expression of an IκBα superrepressor disrupts positive selection of CD8^+^ SP thymocytes, with little effect on the generation of CD4^+^ SP thymocytes [101]. Similarly, genetic ablation of IKKγ or simultaneous deletion of IKKα and IKKβ in the DN or DP thymocyte stage severely reduces the frequency of SP thymocytes, especially CD8^+^ SP thymocytes [99, 102]. IKK/NF-κB appears to be involved in both thymocyte positive selection and SP thymocyte survival [98, 99], although the role of IKK in mediating SP thymocyte survival may also involve NF-κB-independent mechanisms [103]. The level of NF-κB activation, such as the intensity of TCR signaling, appears to be important for thymocyte selection, since excessive NF-κB activation by transgenic expression of constitutively active (CA) IKKβ impairs positive selection of CD4^+^ SP thymocytes and modestly affects the positive selection of CD8^+^ SP [101]. The noncanonical NF-κB pathway regulates thymocyte development by maintaining the normal development of medullary thymic epithelial cells (mTECs), a thymic stromal cell population required for central tolerance [104, 105]. Blocking noncanonical NF-κB activation by genetic ablation of NIK or IKKα in mTECs causes autoimmunity due to impaired generation of thymic Treg cells [1, 105–107]. NIK deletion in T cells has no effect on thymocyte development [108]. Interestingly, however, NIK overexpression in T cells severely impaired thymocyte development, causing lethal autoimmune disorders [109, 110]. However, this function of NIK appears to involve an NF-κB-independent and metabolic mechanism [110].

NF-κB is a vital mediator of peripheral T-cell activation and survival [13] (Fig. 2). The activation of naïve T cells occurs when the TCR is engaged by a specific antigen and the costimulatory molecule CD28 is simultaneously bound by its ligand, B7-1 or B7-2. The canonical NF-κB pathway is activated by the TCR signal and further potentiated by the CD28 costimulatory signal [111, 112]. Genetic evidence suggests a crucial role for the canonical NF-κB pathway in the regulation of T-cell survival, activation, and proliferation [13]. NF-κB is also involved in the regulation of CD4^+^ T-cell differentiation. Upon activation, CD4^+^ T cells differentiate into different subsets of effector T cells, including Th1, Th2, Th17, and Tfh cells [113]. As mentioned above, Th1 and Th17 cells are inflammatory T cells that participate in inflammatory responses to infections and self-triggers. Canonical NF-κB promotes Th1 cell differentiation by regulating TCR signaling as well as the induction of the Th1-polarizing cytokine IL-12 in macrophages and DCs [14, 114]. Both canonical and noncanonical NF-κBs also have a T-cell-intrinsic function in promoting Th1 cell differentiation [108, 115]. The generation of Th17 cells can be induced by several cytokines, including IL-6, TGFβ, IL-1β and IL-23 [116]. The Th17 cells induced by IL-6 and TGFβ are nonpathogenic during inflammation, but they become pathogenic when exposed to the inflammatory cytokines IL-1β and IL-23 [117]. In addition, the T-cell-derived cytokine IL-21 promotes Th17 cell differentiation [118, 119]. The canonical NF-κB pathway promotes Th17 cell differentiation and pathogenesis by inducing the expression of IL-1β, IL-6, and IL-23 in DCs or macrophages and of IL-21 in T cells [5, 120, 121]. In addition, the TCR-induced canonical NF-κB pathway also facilitates Th17 cell differentiation via an intrinsic mechanism [122–124].

Treg cells are a population of Foxp3^+^ immunosuppressive T cells that maintain immune hemostasis and control immune responses to prevent autoimmunity and chronic inflammation [125]. They are divided into natural Treg (nTreg) cells, which are generated in the thymus, and inducible Treg (iTreg) cells, which are generated in the periphery from CD4^+^ naïve T cells [14]. NF-κB regulates the development, stability, and suppressive function of Treg cells [14, 15]. Thymic development of nTreg cells is critically dependent on TCR-stimulated NF-κB activation, since genetic deficiencies in IKKβ or upstream factors in the TCR pathway, including PKCθ, members of the CARMA1-Bcl10-Malt1 complex, and Tak1, impair nTreg development [14, 15]. The expression of an IκBα superrepressor via the T-cell-specific Lck promoter also attenuated nTreg development, whereas increased NF-κB activity via the expression of an active form of IKKβ or the deletion of the IKK-negative regulator CYLD in T cells led to an increased number of thymic Treg cells [126, 127]. Among the NF-κB members, c-Rel is particularly important for Treg development [126, 128, 129]. Mechanistically, c-Rel binds to an enhancer element in the Foxp3 gene locus to drive the expression of Foxp3, which is required for Treg cell development [126, 128]. NF-κB is also required for iTreg generation; genetic ablation of both RelA and c-Rel in naïve T cells blocks the generation of iTreg cells, although deletion of either RelA or c-Rel does not affect iTreg generation [130]. Another role of NF-κB is to maintain the stability and immunosuppressive function of Treg cells [15]. Treg-conditional ablation of IKKβ or its upstream factor Ubc13 impairs the in vivo stability and suppressive function of Treg cells and sensitizes them to acquire Th1- and Th17-like inflammatory cell phenotypes [15, 131]. Consistently, Treg-conditional deletion of the two major canonical NF-κB members, RelA and c-Rel, impairs the hemostasis and function of Treg cells, leading to severe autoimmune inflammation [130].

The noncanonical NF-κB pathway also plays a role in Treg regulation. Mice with T-cell-specific NIK deficiency display a reduced Treg frequency in peripheral lymphoid organs but not in the thymus, suggesting that the noncanonical NF-κB pathway is required for peripheral Treg maintenance but not Treg development [108, 132]. In line with this study, the constitutive expression of NIK in T cells increases the frequency of peripheral Treg cells [109]. Interestingly, however, NIK overexpression impairs the stability and suppressive function of Treg cells, causing autoimmune inflammation [109, 133]. These findings suggest that NIK activation under inflammatory conditions may influence Treg function and thereby promote inflammation. However, it is unclear whether this function of NIK is dependent on the noncanonical NF-κB pathway, since a recent study demonstrated that NIK promotes T-cell metabolism through increasing the steady level of HK2 and that T-cell-conditional deletion of HK2 in NIK-transgenic mice blocks autoimmune phenotypes [110]. However, NF-κB is clearly a master regulator of T-cell immunity and immune tolerance, in addition to its critical role in mediating innate immunity and inflammation.

### Role of NF-κB in inflammatory diseases

**Fig. 3:**
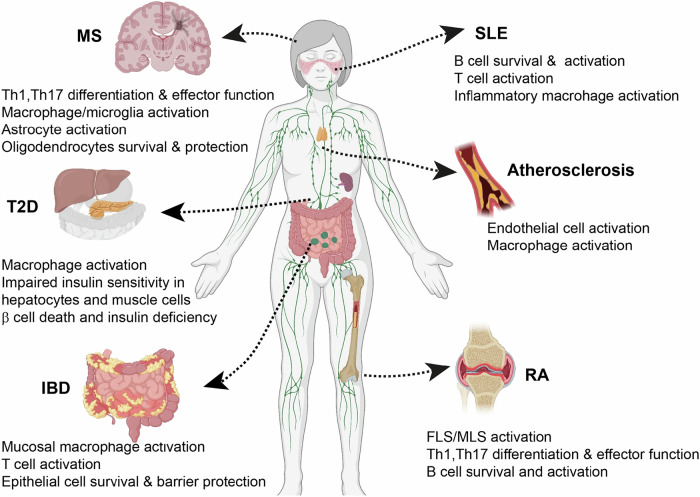
NF-κB in autoimmune and inflammatory diseases. Dysregulated NF-κB is involved in several autoimmune and inflammatory diseases, including rheumatoid arthritis (RA), inflammatory bowel disease (IBD), multiple sclerosis (MS), atherosclerosis, systemic lupus erythematosus (SLE), and type 2 diabetes (T2D), among others. NF-κB contributes to these diseases not only by regulating immune cells, such as macrophages, T cells, and B cells but also by affecting endothelial cells, epithelial cells, astrocytes, oligodendrocytes, hepatocytes, muscle cells and pancreatic β cells. The images were created with BioRender (www.biorender.com)

While NF-κB is required for normal immune and inflammatory responses, aberrant NF-κB activation is a hallmark of autoimmunity and chronic inflammation [134, 135] (Fig. 3).

#### Rheumatoid arthritis

RA is an autoimmune and inflammatory disease characterized by persistent synovitis, systemic inflammation, autoantibody production (particularly rheumatoid factor and anti-citrullinated peptide antibodies), and progressive cartilage and bone damage [136]. The pathogenesis of RA involves multiple cell types, including fibroblast-like synoviocytes (FLSs) and macrophage-like synoviocytes (MLSs) in the synovium, as well as T cells, B cells, and DCs [137]. FLSs and MLSs produce various proinflammatory cytokines, such as IL-1, IL-6, and TNF-α, contributing to synovitis [138]. Notably, FLSs that produce high levels of IL-6 exhibit greater NF-κB1 p50 and RelA binding activity than FLSs that produce low levels of IL-6 [139]. Activated NF-κB has also been observed in a rat adjuvant arthritis model [140], as well as in the vascular endothelium and type A synovial lining cells of RA patients [141]. Consistent with these findings, the inhibition of NF-κB with several traditional Chinese medicines and small-molecule inhibitors has been shown to alleviate RA symptoms [142–146]. Activated NF-κB induces the production of several proinflammatory cytokines, which further recruit and regulate other immune cells, including the CD4^+^ inflammatory Th1 and Th17 cells that play central roles in RA pathogenesis. Significantly increased percentages of IFN-γ-producing Th1 cells and Th17 cells have been found in the peripheral blood of RA patients [147, 148]. Similarly, the serum levels of IL-17, IL-21, and IL-23, cytokines associated with Th17 cell responses, are significantly elevated in RA patients compared with those in osteoarthritis patients, and the frequencies of detectable IL-6, IL-17, and IL-21 are greater in the active RA group than in the inactive RA group [147]. As mentioned above, NF-κB regulates Th1 differentiation either through TCR signaling or by promoting IL-12 production, and NF-κB plays a crucial role in the differentiation and function of Th17 cells.

B cells, major players in autoimmune diseases, are also involved in RA pathogenesis. Several autoantibodies generated by autoreactive B cells—including rheumatoid factor (RF), anti-citrullinated protein, anti-modified citrullinated vimentin, anti-carbamate protein, anti-PAD4, and anti-GPI antibodies—have been detected in RA patients [137]. The NF-κB pathway is involved not only in B-cell development but also in the survival of mature B cells [149]. Conditional deletion of either NEMO or both IKKα and IKKβ in B cells results in a reduced number of immature Igλ-positive B cells [150]. In mice transplanted with hematopoietic stem cells (HSCs) lacking RelA and c-Rel or with B-cell-specific deletions of NEMO or IKKβ, B-cell maturation is blocked at the transitional 1 (T1) stage in the spleen. However, because the blockage is incomplete, some mature B cells still emerge. Nonetheless, the number of mature B cells is significantly reduced, suggesting that the canonical NF-κB pathway plays a crucial role in the maturation and survival of B cells [150, 151]. The noncanonical NF-κB pathway is also indispensable for B-cell maturation and survival, as demonstrated by both mouse models and patients harboring germline mutations in the *Map3k14* (encoding NIK) and *Nfkb2* genes [1]. Inducible deletion of NIK in adult mice causes a reduction in mature B cells and serum antibodies, particularly IgA [152]. Dysregulated noncanonical NF-κB activation due to the loss of upstream negative regulators, such as TRAF2, TRAF3, TBK1, and Otud7b, causes B-cell hyperplasia and aberrant antibody production [1, 153–156]. More evidence suggests the involvement of the noncanonical NF-κB pathway in RA pathogenesis [1, 157]. The synovial tissue of RA patients expresses high levels of noncanonical NF-κB inducers, such as LIGHT, lymphotoxin, and CD40L, which are known to induce the stabilization and accumulation of NIK [1, 158, 159]. Consistently, high levels of the NIK protein are detected in the ECs of inflamed RA synovial tissue [160]. Similarly, patients with early arthritis display increased NIK protein expression in synovial ECs, which is correlated with disease symptoms such as joint swelling, synovitis, and immune cell infiltration [161]. In line with these observations, NIK deficiency renders mice resistant to the induction of inflammatory arthritis [162]. Furthermore, a 3D model of synovial angiogenesis suggested a role for the noncanonical NF-κB pathway in promoting neovascularization [163]. Taken together, both the canonical and noncanonical NF-κB pathways participate in RA pathogenesis, which involves the modulation of synoviocytes, B cells, inflammatory T cells, and multiple other immune cell populations (Fig. 3).

#### Inflammatory bowel disease

IBD, including Crohn’s disease (CD) and ulcerative colitis (UC), is a chronic inflammatory disorder of the gastrointestinal tract. Immunological dysregulation in IBD is characterized by epithelial damage—manifested as abnormal mucus production and defective repair—along with an expansion of inflammation driven by the intestinal flora and the infiltration of various immune cells into the lamina propria, including T cells, B cells, macrophages, DCs, and neutrophils. Failure of immune regulation to control the inflammatory response further exacerbates this condition [164–166].

Activated lamina propria immune cells produce high levels of proinflammatory cytokines, including TNF, IL-1β, and IFN-γ [164, 166], within the local tissue. As a master regulator of proinflammatory cytokines, NF-κB plays a pivotal role in the pathogenesis of IBD (Fig. 3). Polymorphisms in genes encoding NF-κB subunits (especially NF-κB1), NF-κB target genes, and NF-κB regulators are associated with IBD [5, 167]. Increased NF-κB activation has been detected in both the lamina propria and macrophages isolated from inflamed gut specimens of IBD patients [168, 169]. Notably, the level of activated NF-κB is significantly correlated with the severity of intestinal inflammation [170]. In IBD patients, elevated NF-κB expression in mucosal macrophages is associated with an increased capacity of these cells to produce and secrete TNF-α, IL-1, and IL-6 [169, 171]. These cytokines not only drive further stimulation, activation, and differentiation of lamina propria immune cells—thus perpetuating mucosal inflammation—but also contribute to severe extracellular matrix damage and mucosal degradation. This degradation is mediated by TNF-α-induced upregulation of matrix metalloproteinase production [172, 173]. In turn, TNF-a activates NF-κB, further promoting TNF-α production and creating a self-sustaining inflammatory cycle. The proinflammatory role of NF-κB in IBD is further demonstrated by findings that deletion of IKKβ in myeloid cells inhibits both experimental colitis and colitis-associated cancer [174]. Consistently, genetic deficiencies in negative IKK regulators, such as the deubiquitinase CYLD, sensitize mice to intestinal inflammation [175]. CYLD-deficient mice spontaneously develop autoimmunity and colonic inflammation due to aberrant T-cell responses. CYLD-deficient T cells display constitutive NF-κB activity and become hyperresponsive to TCR stimulation, and they induce colonic inflammation in recipient mice upon adoptive transfer [175]. CYLD inactivation in intestinal epithelial cells exacerbates colitis induction by dextran sodium sulfate (DSS), although it does not cause spontaneous colonic inflammation [176]. The noncanonical NF-κB pathway is also involved in IBD pathogenesis. IBD patients display significantly upregulated noncanonical NF-κB signaling in intestinal tissue, which is associated with gastrointestinal inflammation and resistance to anti-TNF-α therapy [177]. Loss of negative regulators of the noncanonical NF-κB pathway, such as NLRP12 and Otud7b, renders mice more susceptible to the induction of colitis [155, 178]. NIK also functions in DCs to regulate mucosal immunity and inflammation, which involves the induction of IL-23 for the maintenance of Th17 cells and type 3 innate lymphoid cells [94].

The role of NF-κB in the regulation of colitis seems to be cell type specific. While NF-κB in myeloid cells is regarded as an explicit proinflammatory mediator in IBD, its role in epithelial cells appears to be anti-inflammatory. Conditional ablation of NEMO or IKKα/IKKβ in intestinal epithelial cells led to the spontaneous development of severe chronic intestinal inflammation in mice, indicating a protective function. NF-κB deficiency results in an increased rate of apoptosis and reduced production of antimicrobial peptides, which impairs epithelial barrier integrity and enhances mucosal inflammation [179]. Furthermore, mice with intestinal epithelial cell-specific deletion of IKKβ presented reduced expression of the epithelial-restricted cytokine thymic stromal lymphopoietin, impaired pathogen-specific Th2 responses, and exacerbated production of proinflammatory Th1 cytokines following parasite infection [180]. However, importantly, the epithelial-specific function of NF-κB should be considered protective rather than anti-inflammatory. In fact, when aberrantly activated in intestinal epithelial cells, NF-κB is still proinflammatory, as observed in mice with epithelial-specific CYLD deletion [176]. Similar phenomena have been observed in studies of the noncanonical NF-κB pathway. Although epithelial cell-specific NIK ablation sensitizes mice to DSS-induced colitis, UC patients are known to have robustly increased epithelial NIK signaling [181]. Moreover, epithelial-specific NIK overexpression also sensitizes mice to colitis induction [181]. These findings suggest that while a physiological level of NF-κB activity in epithelial cells is important for intestinal homeostasis and protection, uncontrolled activation or complete blockade of the NF-κB pathway may increase susceptibility to intestinal injury and colitis. These results suggest crucial and complex functions of NF-κB in IBD pathogenesis and underscore the need for careful design of therapeutic strategies for IBD to avoid unintended adverse effects.

#### Multiple sclerosis

Multiple sclerosis (MS) is a chronic neurological disorder in which the immune system mistakenly attacks healthy cells in the brain and spinal cord, leading to neuroinflammation, demyelination, and neurodegeneration [182, 183]. MS is a complex disease involving interactions between immune cells and central nervous system (CNS) resident cells, including microglia, astrocytes, oligodendrocytes, and neurons. The NF-κB pathway is believed to play a crucial role in the pathogenesis of MS. Genome-wide association analyses have revealed the associations of NF-κB family members, target genes, and regulators with MS [184, 185]. Furthermore, NF-κB is activated in multiple cell types, including T cells, microglia/macrophages, astrocytes, oligodendrocytes, and neurons, in both MS and experimental autoimmune encephalomyelitis (EAE), the most widely used animal model for MS [186–189]. Mice deficient in NF-κB1 p50 exhibit resistance to EAE, as evidenced by a decreased disease incidence, lower clinical scores, and reduced CNS inflammation. Myelin oligodendrocyte glycoprotein (MOG)-specific T cells from these mice display impaired differentiation into Th1 or Th2 effector cells [190]. Similarly, c-Rel-deficient mice are resistant to EAE, which is associated with impaired Th1 and Th17 differentiation [114, 191]. c-Rel promotes proinflammatory T-cell differentiation via both T-cell-intrinsic mechanisms and the regulation of cytokine production in APCs. In line with these findings, T-cell-specific deficiency of IKKβ significantly impairs MOG35-55-specific T-cell responses and provides protection against EAE induction in C57BL/6 mice [192]. These findings indicate that NF-κB activation in T cells may contribute to the pathogenesis of MS and EAE.

Activated microglia/macrophages are hallmarks of MS and EAE, with NF-κB activation observed in these cell types under both conditions. Mice with IκBα ablation specifically in myeloid cells develop a more severe EAE disease course, characterized by increased inflammatory infiltration and CNS demyelination [193]. Correspondingly, IKKβ deficiency in myeloid cells attenuates disease severity, reduces CNS demyelination, and decreases leukocyte infiltration, inflammatory gene expression, and encephalitogenic T-cell activation in EAE mice. Additionally, myeloid cell-specific IKKβ deficiency leads to reduced blood‒brain barrier permeability in the spinal cord during EAE [194, 195]. These findings suggest that NF-κB activation in microglia and macrophages promotes the progression of MS and EAE.

In addition to its role in immune cell regulation, NF-κB also functions in astrocytes and oligodendrocytes to regulate EAE pathogenesis. NF-κB inhibition, specifically in astrocytes, via transgenic expression of an IκBα superrepressor, mitigates EAE severity and improves functional recovery, which is associated with reduced expression of proinflammatory cytokines, chemokines, and adhesion molecules [196]. The reduced EAE disease severity in these transgenic mice is also associated with an increased frequency of CD8^+^CD122^+^ regulatory T cells in the CNS. In contrast to its proinflammatory role in astrocytes and immune cells, NF-κB has a protective role in oligodendrocytes during EAE induction. Selective inhibition of NF-κB in oligodendrocytes via IκBα superrepressor expression exacerbates oligodendrocyte death and myelin loss in young, developing mice that ectopically express IFN-γ, a key proinflammatory cytokine in MS and EAE, in the CNS [197].

The noncanonical NF-κB pathway also plays an important role in the pathogenesis of MS and EAE. A genome-wide analysis identified NIK as a potential MS susceptibility gene [185]. Consistently, NIK-deficient mice are completely protected from MOG-induced EAE, and this function of NIK involves a T-cell-intrinsic mechanism [108, 198, 199]. Although NIK is dispensable for T-cell activation and only partially involved in Th17 cell differentiation, NIK is critical for the pathogenic effector function of Th17 cells [108, 199, 200]. The noncanonical NF-κB member p52 cooperates with the canonical NF-κB member c-Rel to mediate the transcriptional induction of GM-CSF, a key inflammatory cytokine of Th17 cells involved in EAE pathogenesis [200]. Notably, another NF-κB member, RelB, has been shown to play a negative role in regulating Th17 cell function and EAE pathogenesis [201]. It has been proposed that upon activation by OX40, RelB recruits the histone methyltransferases G9a and SETDB1 to the Il17 locus, leading to the deposition of repressive chromatin marks at H3K9 sites. This epigenetic modification suppresses IL-17 expression and alleviates EAE. Conversely, CD4⁺ T cells deficient in RelB exhibit increased IL-17 production and worsened disease severity [201]. Since RelB is activated by both the canonical and noncanonical NF-κB pathways, it remains to be determined whether this function of RelB involves the NIK-dependent noncanonical pathway.

A recent study demonstrated that the noncanonical NF-κB pathway is activated in microglia during EAE induction and mediates the expression of chemokines required for the recruitment of inflammatory T cells to the CNS [202]. This process occurs following initial CNS infiltration with T cells and is required for the subsequent recruitment of more T cells and innate immune cells into the CNS to mediate inflammation during EAE disease progression. Thus, microglia-specific deletion of NIK has no effect on EAE disease onset but attenuates EAE disease progression [202]. Taken together, these findings highlight the multifaceted role of the canonical and noncanonical NF-κB pathways in the pathogenesis of MS and EAE (Fig. 3).

#### Atherosclerosis

Atherosclerosis is a chronic inflammatory disease characterized by lipid accumulation and inflammation in large arteries. It is primarily a lipid-driven process initiated by the accumulation of low-density lipoprotein (LDL) and remnant lipoprotein particles, alongside an active inflammatory response in focal areas of the arteries [203, 204]. Upon stimulation by various factors, vascular ECs express cytokines, chemokines, and cell adhesion molecules that facilitate the recruitment of circulating leukocytes and their migration into the subendothelial layer of the arterial intima [204]. NF-κB activation has been observed in smooth muscle cells, ECs, and macrophages of atherosclerotic lesions [205]. Moreover, hypercholesterolemia induces NF-κB activation in the vessel wall in a pig model of atherosclerosis [206]. The involvement of NF-κB in the pathogenesis of atherosclerosis is further suggested by the finding that EC-specific ablation of NEMO or expression of a dominant-negative IκBα led to a significant reduction in atherosclerotic plaque formation in ApoE^–/–^ mice fed a cholesterol-rich diet [207]. In addition to its role in ECs, NF-κB influences atherosclerosis through macrophages. NF-κB inhibition in macrophages, either via transgenic expression of an IκBα superrepressor or genetic ablation of IKKβ, reduces lipid accumulation, foam cell formation, and atherosclerotic lesion size, which is associated with decreased macrophage activity in inflammatory gene expression, adhesion, migration, and lipid uptake [208, 209]. Correspondingly, myeloid cell-specific deletion of IκBα sensitizes LDL receptor-deficient mice to atherosclerosis, increasing leukocyte adhesion to the luminal side of endothelial cell layers covering atherosclerotic plaques [210]. While these results suggest a role for NF-κB in promoting atherosclerosis in different cell types, a more recent study surprisingly revealed that myeloid cell-specific deletion of IKKβ in LDL receptor-deficient mice leads to increased atherosclerosis during the early phase, characterized by larger and more advanced lesion areas and increased necrosis [211]. However, it is unclear whether this seemingly controversial phenotype is indeed due to NF-κB deficiency, since a recent study demonstrated that IKKβ phosphorylates RIPK1 and inhibits its ability to mediate cell death via an NF-κB-independent mechanism [212]. Furthermore, myeloid cell-specific deletion of RIPK1 limits plaque formation during the early phase of atherosclerosis activation [213]. These findings raise the intriguing question of whether the atherosclerosis-suppressive function of IKKβ is mediated through the inhibition of RIPK1. However, the current evidence generally supports a role for the NF-κB signaling pathway in promoting atherosclerosis in both ECs and macrophages (Fig. 3).

#### Systemic lupus erythematosus

SLE is a chronic autoimmune disease characterized by a wide range of clinical manifestations and a relapsing-remitting course. The pathogenesis of SLE is closely associated with the hyperactivation of various immune cells, including T cells, B cells, and monocytes, which exhibit abnormally methylated and differentially expressed genes [214–218]. Genetic variants in several key components of the TLR/NF-κB signaling axis have been identified in association with lupus nephritis, the leading cause of morbidity and mortality in SLE; these include TLR3, TLR7, TLR9, MYD88, IRAK1, Peli1, and TNFAIP3 [219, 220]. Constitutive activation of NF-κB has also been detected in the B cells of active lupus patients [221]. Furthermore, activation of the TLR/MyD88/NF-κB signaling pathway has been shown to drive the progressive development of multiple SLE-associated phenotypes in mouse models, including splenomegaly, elevated circulating immune complexes, and increased production of pathogenic antinuclear antibodies (ANAs) and anti-dsDNA autoantibodies [222]. These pathological symptoms involve both B cells and myeloid cells. Deregulated activation of the canonical NF-κB member c-Rel in T cells, due to loss of the E3 ubiquitin ligase Peli1, also causes lupus-like autoimmune symptoms in mice, including increased autoantibody production and immune complex deposition in kidney glomeruli [29]. Similarly, NF-κB inhibition protects mice from developing lupus disease. In a nephrotoxic antibody-induced lupus nephritis model, compared with control mice, mice with myeloid cell-specific RelA depletion presented attenuated proteinuria, lower blood urea nitrogen levels, and improved renal histopathology. Rela-deficient myeloid cells also exhibit reduced expression of inflammatory mediators such as IL-1α, IFN-γ, and IL-6 in the kidneys, coupled with a decreased number of classically activated macrophages infiltrating the kidneys of myeloid cell-conditional *Rela*-deficient mice [223]. In addition to the canonical NF-κB pathway, noncanonical NF-κB signaling also plays a role in SLE pathogenesis. A well-known noncanonical NF-κB inducer, BAFF, is closely associated with SLE pathogenesis, and BAFF inhibitors have been actively explored in SLE therapy [1, 224]. Moreover, dysregulated noncanonical NF-κB activation in B cells promotes lupus-like autoimmunity in mouse models [1]. For example, B-cell-conditional ablation of Peli1, an E3 ubiquitin ligase that mediates ubiquitin-dependent NIK degradation in B cells, induces autoantibody production in lupus-like disease via noncanonical NF-κB activation, whereas overexpression of Peli1 inhibits noncanonical NF-κB activation and alleviates lupus-like autoimmune symptoms [225]. Similarly, B-cell-specific deficiency in a deubiquitinase, Otub1, which inhibits ubiquitin-dependent processing and degradation of NF-κB2 p100, causes B-cell hyperplasia and lupus-like autoimmunity [226]. In line with these findings, B-cell-specific deletion of DYRK1, a kinase that phosphorylates TRAF3 and thereby promotes noncanonical NF-κB signaling, inhibits the induction of lupus-like disease [227]. Moreover, inhibition of NIK with a highly selective small-molecule inhibitor alleviated autoimmune symptoms in a mouse model of SLE, as shown by improved survival, reduced renal pathology, and decreased proteinuria scores [228]. In addition to acting on B cells, NIK inhibitors appear to suppress NIK-mediated T-cell activation and inflammatory function as well as the induction of proinflammatory cytokines and chemokines by the NIK inducer TWEAK in kidney epithelial cells [228]. Collectively, these findings underscore the critical role of NF-κB signaling pathways in the pathogenesis of SLE (Fig. 3).

#### Type 2 diabetes

Type 2 diabetes (T2D) is a chronic metabolic disorder characterized by insulin resistance, impaired insulin secretion, and systemic inflammation. T2D is associated with complications such as cardiovascular disease, kidney failure, neuropathy, and retinopathy [229]. Chronic low-grade inflammation is a key contributor to insulin resistance and disease progression, with pathways such as NF-κB playing critical roles [26]. The involvement of NF-κB in T2D was first demonstrated in studies showing that heterozygous deletion of IKKβ (IKKβ^+/−^) in mice protected against insulin resistance during high-fat feeding and in obese Lep ob/ob mice. Similarly, high-dose salicylate treatment improves insulin sensitivity by inhibiting NF-κB [230]. In addition, specific deletion of IKKβ in macrophages protects mice from obesity-induced insulin resistance in skeletal muscle [231]. Consistently, liver-specific expression of CA IKKβ impairs insulin signaling in hepatocytes and muscle cells, increases basal insulin and free fatty acid levels, and leads to systemic insulin resistance and glucose intolerance [232]. Under excessive nutritional conditions, fatty acids induce NF-κB activation via the CBM signaling complex, causing chronic inflammation and rendering hepatocytes resistant to insulin signaling [233]. Genetic deficiency in one of the CBM components, Bcl10, inhibits high-fat diet-induced NF-κB activation and insulin resistance. Moreover, activation of the noncanonical NF-κB pathway has also been linked to impaired insulin secretion. Mice with constitutive NIK activation in pancreatic β cells exhibit defective insulin secretion under diet-induced obesity (DIO) conditions [234]. Additionally, β cell-specific overexpression of NIK (β-NIK-OE) leads to spontaneous diabetes in male mice as early as 10 weeks of age, likely due to insulin deficiency, β cell death, and insulitis [235]. In line with these findings, recent studies based on single-cell RNA sequencing analysis and machine learning models identified NF-κB as an important regulator of immune and metabolic disturbances in T2D [236]. Together, these findings highlight the critical role of both the canonical and noncanonical NF-κB pathways in driving insulin resistance, β-cell dysfunction, and disease progression in T2D patients (Fig. 3).

## NF-κB in cancer

**Fig. 4:**
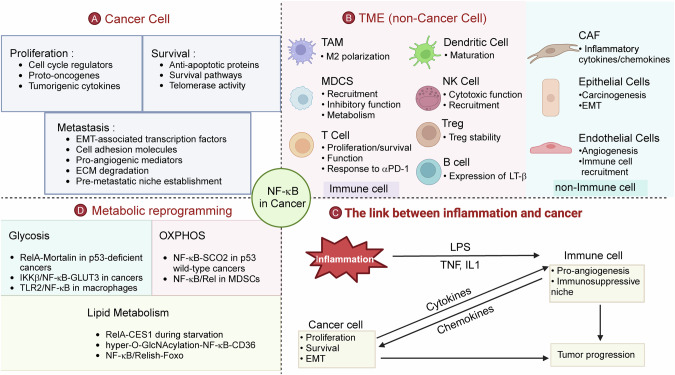
NF-κB in cancer. NF-κB integrates oncogenic, inflammatory, stromal, and metabolic networks during tumorigenesis. **A** Tumor Cell Intrinsic Effects: NF-κB drives cancer cell proliferation, survival and metastasis in many different ways. **B** Tumor microenvironment (TME) remodeling: NF-κB reprograms immune cells (immunosuppressive MDSCs, TAMs, Tregs), activates stromal fibroblasts (ECM remodeling via CAFs), and promotes angiogenesis (endothelial activation) in the TME. **C** Inflammation-Cancer Crosstalk: Chronic inflammation (IL-6/STAT3, TNF-α/NF-κB loops) activates NF-κB in tumor and immune cells, sustaining oncogenic signaling, immune evasion, and malignant progression. **D** Metabolic reprogramming: NF-κB enhances glycolysis, suppresses oxidative phosphorylation (OXPHOS), and promotes lipid metabolism in the TME via different mechanisms. The images were created with BioRender (www.biorender.com)

Cancer development is a multistage process encompassing tumor initiation, promotion, and progression, driven by genetic mutations, epigenetic dysregulation, and dynamic interactions within the tumor microenvironment (TME). During cancer initiation, environmental carcinogens, chronic inflammation, and spontaneous DNA damage promote oncogenic mutations (e.g., KRAS and TP53) and genomic instability. In the promotion phase, hyperproliferation of transformed cells is fueled by inflammatory cytokines (e.g., IL-6 and TNF-α) and growth factors, whereas evasion of apoptosis and immune surveillance enables clonal expansion. Finally, progression involves the acquisition of invasive and metastatic traits through epithelial‒mesenchymal transition (EMT), angiogenesis, and metabolic adaptation [237]. The transcription factor NF-κB, a master regulator of inflammation and stress responses, has emerged as a central orchestrator of these processes, linking intrinsic oncogenic signaling to extrinsic inflammatory cues and TME remodeling. During tumorigenesis, NF-κB fuels genomic instability through reactive oxygen species (ROS), reactive nitrogen intermediates (RNIs) and mutagenic enzymes while enabling survival via antiapoptotic proteins (e.g., Bcl-2 and XIAP) and proliferation through cyclins D1 and MYC. It directly drives metastasis by inducing EMT via TWIST1 and SNAIL while promoting angiogenesis through VEGF and IL-8. Critically, NF-κB bridges chronic inflammation and malignancy by sustaining cytokine loops (e.g., IL-6/STAT3 and TNF-α/NF-κB) that amplify oncogenic signaling [238]. Within the TME, NF-κB reprograms immune cells to an immunosuppressive phenotype, activates stromal fibroblasts to remodel the extracellular matrix, and hijacks ECs to promote vascular permeability. Furthermore, it rewires tumor metabolism by enhancing glycolysis (Warburg effect), lipid synthesis, and oxidative phosphorylation (OXPHOS) suppression, ensuring biosynthetic adaptability (Fig. 4). Given its multifaceted roles in cancer biology, spanning cell-intrinsic oncogenesis, TME modulation, and metabolic reprogramming, a comprehensive understanding of the role of the NF-κB signaling pathway in cancer is crucial for developing novel therapeutic strategies.

### NF-κB as a driver of tumorigenesis

In cancer, NF-κB is persistently activated by diverse stimuli, including cytokines, pathogens, DNA damage, hypoxia, and oncogenic mutations, which override normal regulatory feedback mechanisms. Such deregulated activation enables NF-κB to transcriptionally reprogram cancer cells, promoting proliferation by upregulating cell cycle drivers, enhancing survival through antiapoptotic proteins, and facilitating metastasis via genes governing EMT and extracellular matrix remodeling, thus contributing to tumor progression (Fig. 4).

#### Proliferation

NF-κB plays a pivotal role in driving cancer cell proliferation through its ability to promote cell cycle progression and suppress apoptosis. Central to its pro-proliferative function is the transcriptional regulation of key cell cycle regulators, including cyclin D1, cyclins D2/D3, CDK4/6, and c-Myc, which orchestrate the G1/S phase transition and sustain uncontrolled cell division [239]. In breast cancer, NF-κB activation via RANK- or PAK5 (P21cdc42/rac1-activated kinase 5)-mediated phosphorylation of RelA enhances cyclin D1 expression by facilitating NF-κB nuclear translocation and binding to the cyclin D1 promoter, thereby accelerating cell cycle progression [240–242]. This regulatory axis is further supported by evidence showing that pharmacological inhibition of NF-κB (e.g., via simvastatin) downregulates cyclin D1 and CDKs, inducing cell cycle arrest at G0/G1 and apoptosis [243]. In addition to cyclins, NF-κB intersects with oncogenic signaling pathways such as the PI3K-AKT-mTOR pathway, where cytokine- or growth factor-induced AKT activation primes IKKα-dependent NF-κB activation, fostering survival and proliferation [244]. Additionally, NF-κB sustains proliferative signals by acting downstream of proto-oncogenes such as Ras and c-Myc, with gain-of-function mutations in Ras superfamily members sustaining NF-κB activity in cancers such as lung adenocarcinoma, where IKKβ/NF-κB inhibition suppresses tumorigenesis [245]. In addition to targeting IKKβ for activation, Ras family members promote the transcriptional activity of NF-κB [246, 247]. NF-κB also amplifies tumorigenic cytokine networks (e.g., IL-6, TNF, and IL-1β) and growth factor receptors (EGFR and HER2), which feed forward to activate NF-κB in a self-reinforcing loop. For example, HER2-driven breast cancer progression relies partly on NF-κB activation [248], whereas EGFR expression itself is transcriptionally regulated by NF-κB [249], highlighting its multifaceted role in growth signaling. Emerging evidence underscores the involvement of noncoding RNAs, such as lncRNA NEAT1 (nuclear enriched abundant transcript 1) [250] and miR-505 [251], in modulating NF-κB-mediated proliferation, further illustrating the complexity of its regulatory network. This multifaceted regulatory network positions NF-κB as a central orchestrator of cancer cell proliferation, with its constitutive activation creating a permissive environment for unchecked growth and tumor progression.

#### Survival

NF-κB is a central mediator of cancer cell survival, primarily through its robust antiapoptotic activity, which enables tumor cells to evade programmed cell death and resist therapeutic interventions. This transcription factor orchestrates survival by upregulating a repertoire of antiapoptotic proteins, including inhibitors of apoptosis proteins (IAPs), such as c-IAP1, c-IAP2, XIAP, and c-FLIP, which directly suppress caspase-8 activity and inhibit extrinsic apoptotic signaling [20, 21, 252]. Moreover, NF-κB transcriptionally induces the expression of Bcl-2 family members, including Bcl-2 and Bcl-xL, which block mitochondrial apoptosis by preventing BAX/BAK oligomerization, cytochrome c release, and apoptosome formation [253]. In breast cancer, NF-κB-driven overexpression of Bcl-2 enhances chemoresistance and metastatic potential, whereas pharmacological suppression of NF-κB (e.g., via chrysophanol) downregulates Bcl-2 and cyclin D1, restoring apoptotic sensitivity [254]. NF-κB further antagonizes apoptosis by modulating survival pathways such as the PI3K-AKT axis, where it transcriptionally represses PTEN through Snail activation [255] or miR-130b/301b-mediated USP13 suppression [256], thereby amplifying AKT signaling and sustaining survival. Additionally, NF-κB inhibits p53-dependent apoptosis by promoting p53 polyubiquitylation and degradation, counteracting proapoptotic signals triggered by oncogenic Ras mutations [257]. The role of NF-κB in therapy resistance is exemplified by the suppression of TNF- or TRAIL-induced apoptosis via the upregulation of c-FLIP, which is critical for neutralizing death receptor signaling [258]. NF-κB also suppresses apoptosis via epigenetic mechanisms. For example, constitutive activation of the canonical NF-κB member RelA downregulates the expression of the H3K36 trimethylase NSD1 (also called KMT3B), thereby restricting the expression of the proapoptotic factor BIM [259]. Another function of NF-κB is to increase telomerase activity via transcriptional induction of the telomerase catalytic subunit telomerase reverse transcriptase (TERT) [260, 261], a mechanism that inhibits apoptosis and enables replicative immortality of cancer cells [262]. This function involves cooperation between the noncanonical NF-κB and ETS families of transcription factors [261]. Collectively, although the pro-apoptotic roles of NF-κB have also been implicated in some situations, such as the induction of certain death receptors [263, 264], the prevailing function of NF-κB in cancer remains anti-apoptotic, which underpins tumor progression and therapeutic failure. Targeting NF-κB-mediated survival mechanisms, such as treatment with survivin inhibitors (YM155) combined with TRAIL or γ-radiation, has shown promise in sensitizing resistant cancers to apoptosis, highlighting its therapeutic relevance [265]. Overall, the ability of NF-κB to integrate diverse antiapoptotic signals positions it as a linchpin in cancer cell survival and a critical barrier to effective treatment.

#### Metastasis

The NF-κB signaling pathway plays a pivotal role in modulating multiple molecular mechanisms underlying cancer metastasis, a process responsible for the majority of solid tumor-related deaths [266]. Central to its function is the regulation of EMT, a critical event in the initiation of metastasis. NF-κB directly modulates EMT-associated transcription factors, including TWIST1, SNAIL, ZEB1, and CDH2 [267–270], which drive the loss of epithelial characteristics and enhance migratory potential. For example, in breast cancer, the TNF-α/NF-κB/TWIST1 signaling axis promotes EMT, suggesting that therapeutic targeting of this axis may impede metastasis [267]. Similarly, the inhibition of NF-κB in pancreatic cancer suppresses the expression of EMT markers (SNAI1, SNAI2, and VIM) and reduces invasive capacity, which is reversed by IKK activation [269].

Metastatic progression further relies on NF-κB-mediated induction of cell adhesion molecules, such as vascular cell adhesion molecule-1 (VCAM-1), endothelial leukocyte adhesion molecule (ELAM-1), and ICAM-1, which facilitate the tumor cell‒endothelial interactions critical for extravasation. Additionally, NF-κB upregulates proangiogenic mediators such as VEGF, IL-8, and MMP-9, which promote neovascularization and matrix remodeling [271]. In prostate cancer cells, constitutive NF-κB activation enhances VEGF and IL-8 expression, promoting endothelial cell recruitment and angiogenesis [272]. MMP-9, a key matrix metalloproteinase regulated by NF-κB, not only degrades the extracellular matrix (ECM) but also liberates bioactive VEGF, amplifying angiogenic switching [273].

NF-κB also governs the degradation of the ECM through the upregulation of urokinase-type plasminogen activator (uPA) and its receptor (uPAR), which initiates proteolytic cascades essential for invasion [274]. In pancreatic cancer cells, RelA transcriptionally activates uPA, linking constitutive NF-κB activity to metastatic potential [275]. Conversely, metastasis suppressors such as BRMS1 inhibit NF-κB by blocking IκBα phosphorylation and suppressing uPA, underscoring the pathway’s dual regulatory potential [275]. Emerging evidence highlights the role of NF-kB in the epigenetic regulation of metastasis through mechanisms such as DNA methylation. Notably, TNF-α-induced phosphorylation of RelA at serine 276 in certain cancer cells facilitates the recruitment of DNA methyltransferase 1 (Dnmt1) to tumor suppressor genes (e.g., BRMS1). Assembly of the RelA/Dnmt1 complex at the BRMS1 promoter region results in gene hypermethylation and transcriptional repression, which are associated with a dramatic increase in tumor metastasis [276]. NF-κB also shapes the premetastatic niche; for example, serum amyloid A3 (SAA3) activates NF-κB in lung epithelial and myeloid cells through triggering TLR4 signaling, establishing an inflammatory microenvironment conducive to metastasis [277].

In summary, NF-κB serves as a master regulator of metastasis through EMT induction, adhesion/angiogenesis modulation, ECM degradation, and niche preparation. Targeting this pathway, either directly or via downstream effectors, holds promise for curbing metastatic dissemination.

### NF-κB as a link between inflammation and cancer

NF-κB, a pivotal transcription factor in inflammatory signaling, serves as a molecular bridge connecting chronic inflammation to cancer initiation and progression (Fig. 4). Its activation in response to pathogens, tissue damage, or proinflammatory cytokines such as tumor necrosis factor (TNF)-α and IL-1β drives the expression of genes critical for immune responses, cell survival, and proliferation. Persistent NF-κB activation, however, fosters a tumor-promoting microenvironment by orchestrating inflammatory cascades that facilitate carcinogenesis, immune evasion, and metastasis.

Chronic inflammation, driven by persistent infections (e.g., *H. pylori*, HBV, and HCV), autoimmune disorders, or environmental insults (e.g., tobacco and obesity), which increase cancer risk [237, 278–280], is associated with sustained NF-κB activation in both immune and epithelial cells. This activation is mediated by proinflammatory cytokines, PAMPs, and DAMPs via PRRs such as TLRs and NLRs [237, 281]. Once activated, NF-κB orchestrates a transcriptional program that fuels carcinogenesis through multiple mechanisms. In immune cells, NF-κB drives the production of protumorigenic cytokines (e.g., TNFα, IL-6, IL-1, and IL-17A) and chemokines (e.g., CXCL1 and CCL2), establishing a chronic inflammatory microenvironment conducive to DNA damage, genomic instability, and epithelial cell transformation. For example, in colitis-associated cancer (CAC), NF-κB activation in myeloid cells drives IL-6 production, which subsequently activates STAT3 in intestinal epithelial cells (IECs), promoting the survival and proliferation of premalignant cells [174, 282]. Similarly, chronic activation of NF-κB in hepatocytes contributes to the development of cholestatic hepatitis followed by hepatocellular carcinoma in a mouse model with genetic deficiency in multidrug resistance 2 (Mdr2) [283].

NF-κB drives tumor-promoting inflammation through multiple interconnected mechanisms. First, it induces the production of proinflammatory cytokines such as TNF-α, IL-6, and IL-1β, as well as chemokines such as CXCL1 and CCL2, which recruit immune cells, stimulate angiogenesis, and enhance cancer cell proliferation. For example, IL-6, a key NF-κB target, activates the STAT3 and MAPK pathways in tumor cells, promoting survival under hypoxic or nutrient-deprived conditions [284]. Second, NF-κB upregulates antiapoptotic genes (e.g., BCL-2 and XIAP), enabling cancer cells to evade programmed cell death. In pancreatic cancer, nitric oxide (NO)-mediated IL-1β secretion establishes a paracrine loop that activates NF-κB, conferring chemoresistance [285]. Third, NF-κB promotes EMT and metastasis by regulating transcription factors such as Twist and Slug. In breast cancer, tumor-associated macrophages (TAMs) secrete IL-11, which activates NF-κB and STAT3 to drive invasive phenotypes [286]. While uncontrolled NF-κB activation leads to tumor-promoting inflammation, physiological NF-κB function in some cell types plays a protective role. For example, genetic ablation of NEMO, a component of the IKK complex, in liver parenchymal cells causes inflammation due to hepatocyte apoptosis, leading to spontaneous development of hepatocellular carcinoma [284]. In established tumors, NF-κB may also promote immunosuppression by increasing the levels of immunosuppressive cytokines (e.g., IL-10 and TGF-β) and recruiting myeloid-derived suppressor cells (MDSCs), thereby promoting tumor progression. For example, NF-κB activation in hepatocytes through transgenic expression of an upstream inducer, cell cycle-related kinase (CCRK), induces expression of the chemokine CXCL1 and thereby recruits polymorphonuclear MDSCs (PMN-MDSCs) to promote liver metastasis [287]. Together, these findings highlight a central role for NF-κB in shaping a protumorigenic microenvironment through the modulation of inflammatory signaling.

While NF-κB predominantly acts as a tumor promoter, its role is context dependent. Acute NF-κB activation in immune cells can exert antitumor effects by enhancing cytotoxic T-cell responses [288]. Conversely, chronic activation of epithelial or stromal cells promotes malignancy. For example, IKKβ deletion in myeloid cells exacerbated melanoma growth via altered cytokine profiles, whereas its loss in enterocytes reduced the incidence of CAC despite increased inflammation [174]. This duality complicates therapeutic targeting, as systemic NF-κB inhibition may disrupt antitumor immunity.

In summary, NF-κB lies at the nexus of inflammation and cancer, driving tumorigenesis through cytokine networks, immune modulation, and genomic instability. Its dual roles underscore the complexity of therapeutic intervention, necessitating nuanced approaches to disrupt protumorigenic signaling while preserving immune surveillance. Future research must elucidate context-specific NF-κB mechanisms to harness its potential as a therapeutic target.

### NF-κB and the TME

The TME is an interactive system comprising immune cells, stromal cells and the ECM that collectively influences tumor progression, metastasis, and therapeutic resistance. As a master regulator of inflammation and immune responses, NF-κB plays a pivotal role in shaping the TME by modulating both immune and stromal components (Fig. 4). The multifaceted contributions of NF-κB to tumor-associated immune and stromal cell functions are highlighted, emphasizing its context-dependent pro- or antitumorigenic effects.

#### Role of NF-κB in immune cells

##### TAMs

TAMs, the most abundant immune cells in the TME [237], exhibit phenotypic plasticity between the proinflammatory M1 and immunosuppressive M2 states [289]. NF-κB activation in TAMs drives M2 polarization through the transcriptional regulation of anti-inflammatory mediators such as IL-10 and TGF-β, which suppress cytotoxic T-cell activity and promote angiogenesis via VEGF secretion [290]. NF-κB1 p50 is particularly critical in sustaining the M2 phenotype; its inhibition reprograms TAMs toward an M1-like state, characterized by the production of TNF-α, IL-12, and iNOS, enhancing tumor cell killing [289, 291–293]. Paradoxically, NF-κB also mediates early proinflammatory responses in TAMs via cytokines such as IL-1β and IL-6, which fuel tumorigenesis by promoting cancer cell survival and proliferation [284, 294–296]. Cycling hypoxia in the TME further amplifies NF-κB activity through JNK/RelA signaling, driving sustained proinflammatory gene expression in M1-like TAMs [297]. However, chronic NF-κB activation in advanced tumors shifts TAMs toward immunosuppression via the upregulation of PD-L1 and Arginase-1, highlighting the temporal duality of NF-κB in TAM biology.

##### DCs

DCs bridge innate and adaptive immunity by presenting tumor antigens to T cells. In the TME, DCs, particularly conventional type 1 DCs (cDC1s), are vital for antitumor immunity because of their ability to cross-present tumor antigens to activate CD8+ T cells [298]. The function of DCs is dynamically regulated in the TME by cytokines, metabolites and intracellular signaling pathways. For example, a recent study identified a cluster of DCs coexpressing immunoregulatory molecules (e.g., PD-L1, PD-L2 and CD200) and maturation-associated molecules (e.g., CD40, CCR7, and IL-12) [299]. This cluster, referred to as mature DCs enriched with immunoregulatory molecules (mregDCs), displays a low level of immunostimulatory functions, but their T-cell-activation functions can be enhanced by the induction of IL-12 expression via IL-4 blockade [299]. Both the canonical and noncanonical NF-κB pathways play important roles in regulating the antitumor functions of DCs. Canonical NF-κB activation in CCR7^+^ cDC1s enhances chemokine secretion (e.g., CCL19/21) and antigen cross-presentation, promoting CD8^+^ T-cell infiltration and antitumor responses [300]. Similarly, the noncanonical NF-κB pathway is required for the functions of DCs in cross-presentation, CD40 signaling, and IL-12 production [92, 93]. For example, NIK deletion in DCs abolishes CD8^+^ T-cell activation in melanoma models, underscoring its therapeutic relevance [93].

##### MDSCs

NF-κB signaling plays a central role in regulating the immunosuppressive functions of MDSCs within the TME. Canonical NF-κB signaling, triggered by pathways such as TLR2/4-MyD88 and TNFR/TNFR2, enhances MDSC immunosuppression by increasing IL-10 and TGF-β production, thereby dampening T-cell responses and fostering tumor progression [301–303]. In pancreatic ductal adenocarcinoma, NF-κB is activated by the pro-oncogenic protein cysteine-rich intestinal protein 1 (CRIP1), which results in the transcriptional induction of the chemokines CXCL1 and CXCL5 and the recruitment of MDSCs to promote immunosuppression [304]. CXCL inhibition blocks MDSC recruitment and enhances CD8^+^ T-cell activation, restoring immunotherapy sensitivity [304]. In addition, c-Rel deletion reprograms the metabolism of MDSCs, reducing OXPHOS and enhancing glycolysis, which impairs their tumor-promoting capacity [305]. Furthermore, IL-1β-driven NF-κB activation in MDSCs suppresses antitumor immunity, leading to tumor proliferation [306].

##### NK cells

NK cells eliminate tumor cells via perforin and granzyme B [73, 74], whose expression is directly regulated by NF-κB [307, 308]. Canonical NF-κB activation in NK cells, triggered by activating receptors (e.g., NKG2D, CD226) [309–312] or cytokines such as IL-18 via MyD88-dependent pathways [313–315], promotes NK cell activation and cytotoxicity. Studies have demonstrated that NF-κB activation enhances NK cell cytotoxicity in models such as those of pancreatic cancer, where polysaccharides stimulate TLR4/MAPK/NF-κB signaling to increase tumor-killing capacity [316]. NF-κB also mediates tumor cell expression of chemokines, such as CXCL12, which recruit NK cells, as shown in a head and neck squamous cell carcinoma (HNSCC) model where CHMP2A (chromatin-modifying protein/charged multivesicular body protein) ablation activates NF-κB and amplifies chemokine secretion [317].

##### T lymphocytes

NF-κB signaling is indispensable for the differentiation, activation, and effector functions of T lymphocytes. During T-cell development, canonical NF-κB activation via the CARMA1-Bcl10-MALT1 (CBM) complex, triggered by TCR-peptide-MHC interactions, governs both positive and negative selection [318]. Within the TME, NF-κB critically regulates CD8^+^ T-cell proliferation, survival, and cytotoxic activity. Genetic amplification of NF-κB signaling—through constitutive IKKβ activation or deletion of ubiquitin-modifying enzyme A20 (TNFAIP3)—enhances tumor-specific IFN-γ production, augments cytotoxic function, and promotes tumor rejection in preclinical models [319–321]. Conversely, impaired NF-κB activity in CD8^+^ T cells, as observed in renal cell carcinoma, is correlated with poor antitumor responses [322, 323]. The NF-κB pathway may also intersect with immune checkpoint dynamics, since CD28 engagement is critical for the efficacy of anti-PD-1 therapy in mice, and TCR/CD28 costimulation activates NF-κB via the CBM complex [324].

In conventional CD4^+^Foxp3^−^ T cells (Tcon), NF-κB subunits regulate differentiation into distinct helper subsets (Th1, Th2, Th17, Th9) [114, 325–330]. Notably, c-Rel, a canonical NF-κB subunit, is critical for Tconv-mediated antitumor immunity, as demonstrated by its essential role in enhancing anti-PD-1 efficacy in melanoma models [331]. Paradoxically, NF-κB also governs the immunosuppressive functions of Treg cells, which are key drivers of tumor immune evasion. RelA contributes to Treg stability and suppressive activity, with c-Rel specifically required for thymic Treg development [332]. Strikingly, genetic or pharmacological inhibition of c-Rel destabilizes the Treg identity, triggering IFN-γ production and converting it into antitumor effectors, thereby suppressing tumor growth and synergizing with checkpoint blockade [333]. NF-κB also orchestrates the epigenetic reprogramming of CD4^+^ T cells in tumors. Glucocorticoid-induced TNFR-related protein (GITR) ligation shifts activated CD4^+^ T cells from Foxp3^+^ iTregs to the Th9 lineage, enhancing antitumor immunity. Mechanistically, GITR upregulates p50 to recruit histone deacetylases to the Foxp3 locus to produce a ‘closed’ chromatin structure while simultaneously activating STAT6 to promote Il9 transcription via recruitment of the histone acetyltransferase p300. This dual chromatin remodeling axis underscores the role of NF-κB in dynamically balancing immunosuppressive and effector T-cell programs within the TME [334].

##### B lymphocytes

B cells in the TME play different roles. NF-κB is critical for B-cell maturation; combined c-Rel and NF-κB1 (p105/p50) deficiency disrupts germinal center formation and humoral immunity [335]. In the TME, the local environment of cancer patients presents an increased presence of switched memory B cells and antibody-secreting B cells, suggesting a potential regulatory role of B cells in tumor progression via the modulation of cytokines, including inflammatory factors [336]. CXCL13-mediated NF-κB activation in B cells induces LT secretion, which enhances leukocyte infiltration and metastatic potential via the IKKα–BMI1 axis, particularly in androgen-deprived prostate cancer [337–339]. In addition, IgA^+^ plasmocytes express PD-L1 and IL-10, directly inhibiting CTLs [340]. Intriguingly, human melanoma secretomes upregulate NF-κΒ-dependent chemokines (e.g., CCL3/CCL4) and costimulatory molecules in B cells, which is correlated with improved patient survival and an anti-PD-1 response [341].

#### Role of NF-κB in stromal cells

##### Cancer-associated fibroblasts (CAFs)

CAFs are pivotal stromal components within the TME and are activated by tumor-induced alterations in tissue architecture, TGF-β signaling, or hypoxia [339, 342, 343]. Once activated, CAFs adopt a proinflammatory transcriptional profile characterized by the secretion of proinflammatory cytokines (e.g., TNF, IL-1β, and IL-6), chemokines (e.g., CXCL12 and CXCL1/2), and growth factors (e.g., VEGF), which collectively drive tumorigenesis, metastasis, and angiogenesis through ECM remodeling and immune cell recruitment, partly through NF-κB-dependent mechanisms [342–344]. For example, CAF-derived CXCL12 serves as an organotropic factor to mediate the metastasis of CXCR4^+^ cancer cells [345], and CAF-derived IL-11 drives colorectal cancer metastasis through STAT3 activation in malignant cells [346]. Furthermore, complement signaling-mediated activation of NF-κB in CD10⁺GPR77⁺ CAFs drives cancer stem cell (CSC) enrichment and chemoresistance through IL-6 and IL-8 paracrine secretion [347]. In mouse models of colon carcinoma, IL-6 and GM-CSF secreted by CAFs drive monocyte differentiation toward M2-like TAMs [348], whereas CXCL13 recruits lymphotoxin-expressing B cells to increase prostate cancer aggressiveness [337, 339]. In breast cancer, miR-221 inhibition suppresses NF-κB activity in CAFs, curtailing protumorigenic cytokine secretion [349]. Intriguingly, gastric cancer cells influence the continuous activation of the NF-κB signaling pathway in CAFs by secreting tumor exosomes containing PKM2, thus inducing abnormal metabolism and inflammatory activation [350]. Paradoxically, NF-κB signaling in CAFs has dual regulatory roles: IKKβ activation suppresses hepatocyte growth factor (HGF)-mediated intestinal tumorigenesis via SMAD7 and SMURF1 induction [351] but promotes colitis-associated cancer through IL-6 overproduction [352]. This functional dichotomy underscores the context-dependent influence of the pathway on stromal–tumor interactions.

##### Epithelial cells and endothelial cells

Within the TME, epithelial cells undergo phenotypic plasticity that drives oncogenesis and stromal reprogramming. NF-κB activation in these cells synergizes with Wnt signaling to induce dedifferentiation of nonstem epithelial cells into tumor-initiating populations, a critical step in early carcinogenesis [353]. This crosstalk is amplified by IL-6, which is secreted predominantly by myeloid cells and activates STAT3 and β-catenin nuclear translocation in epithelial cells, fostering colorectal cancer progression [354]. NF-κB further governs EMT by upregulating the expression of transcription factors such as Twist, Snail, and ZEB-1/2, which disrupt epithelial integrity and increase invasiveness [355–357]. Matrix metalloproteinases (MMPs), particularly the EMT effector MMP-9, are regulated by NF-κB-mediated expression of myosin light chain kinase (MLCK) and IL-6 in intestinal tumors [358]. Conversely, RelA silencing downregulates MMP-9 and inhibits proliferation and invasion in human esophageal squamous cell cancer (ESCC) [359]. This intricate regulatory mechanism of NF-κB underscores its integral role in the modulation of EMT, further indicating its contribution to cancer progression.

ECs similarly exploit NF-κB to shape angiogenic and immunosuppressive niches. Fluid shear stress and platelet-activating factor (PAF) induce MMP-9 expression in ECs via NF-κB, accelerating tumor vascularization and transendothelial metastasis [360, 361]. VEGF receptors (VEGFR1/2) are overexpressed in tumor-associated ECs, with DHA-mediated VEGFR2 suppression linked to the NF-κB motif [362–364]. The noncanonical NF-κB pathway functions in ECs to mediate the induction of CXCL12, a chemokine critical for angiogenesis and immune cell recruitment [365–368]. Additionally, EC-derived MCP-1, a chemoattractant for monocytes, T lymphocytes, and basophils, requires coordinated NF-κB and AP-1 signaling for maximal induction by cytokines such as IL-1β [369].

**Fig. 5:**
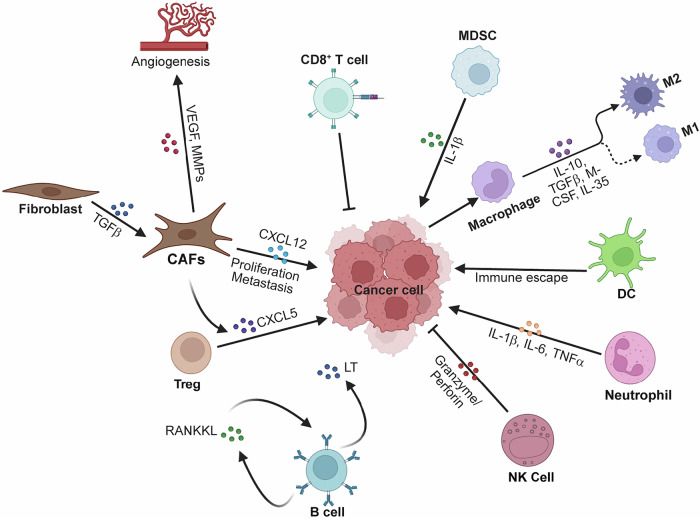
The NF-κB pathway regulates the tumor microenvironment. The NF-κB signaling pathway is closely associated with the different constituents of the tumor microenvironment. NF-κB induces chemokine secretion across stromal, tumor, and immune cells while simultaneously enhancing cytokine transcription through its activation in these cellular compartments. These coordinated molecular events collectively facilitate tumor progression and metastasis

In summary, NF-κB is a central architecture of the TME that modulates immune evasion, angiogenesis, and stromal activation through cell type-specific mechanisms. The NF-κB signaling pathway is closely associated with the different constituents of the TME (Fig. 5). Its dual roles—proinflammatory in early tumors and immunosuppressive in advanced stages—necessitate context-dependent therapeutic targeting.

### NF-κB in metabolic regulation within the TME

The TME is a dynamic ecosystem where cancer cells interact with stromal and immune components under conditions of metabolic stress. NF-κB plays a central role in reshaping tumor metabolism to meet the biosynthetic and bioenergetic demands of rapid proliferation, immune evasion, and therapy resistance. This transcription factor family orchestrates metabolic adaptations across glycolysis, OXPHOS, and lipid metabolism, establishing a symbiotic relationship between tumor progression and immunosuppression (Fig. 4).

#### NF-κB in glycolysis and OXPHOS

Glycolytic reprogramming, a hallmark of cancer, enables tumor cells to thrive in the hypoxic, nutrient-depleted TME. NF-κB directly fuels the Warburg effect through multiple mechanisms. In p53-deficient tumors, RelA translocates to mitochondria via interaction with the heat shock protein Mortalin and assistance by STAT3, where it binds to mitochondrial DNA (mtDNA) to induce mtDNA instability and thereby represses OXPHOS and ATP production, locking cells into glycolysis [370, 371]. However, in p53 wild-type cancers, NF-κB promotes OXPHOS by upregulating p53-dependent expression of SCO2 (synthesis of cytochrome c oxidase 2) [372]. Additionally, the IKKβ/NF-κB axis upregulates the glucose transporter GLUT3, enhancing glucose uptake and glycolytic flux [373]. A positive feedback loop between glycolysis and the IKK/NF-κB pathway supports oncogenic transformation driven by H-Ras [374]. Wang et al. reported that under low glucose conditions, glutamate dehydrogenase 1 (GDH1) is phosphorylated at serine 384 and interacts with RelA and IKKβ. GDH1-produced α-ketoglutarate (α-KG) directly binds to and activates IKKβ and NF-κB signaling, which promotes glucose uptake and tumor cell survival by upregulating GLUT1, thereby accelerating gliomagenesis [375]. Notably, IKKβ also acts as a metabolic sensor in an NF-κB-independent manner; under low-glutamine conditions, it phosphorylates and inhibits the activity of a major glycolysis driver, 6-phosphofructo-2-kinase/fructose-2,6-biphosphatase isoform 3 (PFKFB3), thereby inhibiting aerobic glycolysis and redirecting glucose-derived carbons to the TCA cycle and the pentose phosphate pathway to reduce glutamine dependence for the generation of TCA cycle intermediates and the suppression of ROS [376]. Thus, simultaneous inhibition of IKKβ activity and glutamine metabolism is capable of synergistic killing of cancer cells.

NF-κB-driven metabolic reprogramming extends beyond cancer cells to shape immune responses. Tumor-derived exosomes hijack TLR2/NF-κB signaling in macrophages, suppressing OXPHOS and increasing lactate production, which drives PD-L1 expression and immunosuppression [377]. In MDSCs, NF-κB drives OXPHOS-dependent immunosuppression. C-Rel-deficient MDSCs shift toward glycolysis, impairing their immunosuppressive function and enhancing CD8^+^ T-cell infiltration. This phenotype is rescued by C/EBPβ overexpression, which restores OXPHOS and the expression of proinflammatory cytokines [305]. Similarly, lactate, derived from glycolytic tumor cells, activates NF-κB in CAFs, inducing HGF production to drive therapy resistance [378]. Lactate can also enter ECs through the monocarboxylate transporter MCT-1, trigger the phosphorylation/degradation of IκBα, and then stimulate the autocrine NF-κB/IL-8 (CXCL8) pathway, which drives tumor angiogenesis. Importantly, tumor-derived lactate undermines CD8^+^ T-cell cytotoxicity by suppressing pyruvate carboxylase and disrupting TCA cycle anaplerosis. Restoring metabolic function through pyruvate dehydrogenase targeting enhances succinate secretion, which activates succinate receptor signaling to sustain cytotoxic activity in lactate-rich TMEs [379]. Thus, NF-κB drives tumor progression by coupling cancer-intrinsic glycolytic/OXPHOS reprogramming with immunosuppressive immunometabolism in the TME to sustain both tumor survival and immune evasion.

#### NF-κB and lipid metabolism in the TME

Lipid metabolic reprogramming supports membrane synthesis, energy storage, and signaling in cancer. NF-κB regulates lipid catabolism and synthesis to adapt to nutrient stress. In colorectal cancer (CRC), RelA upregulates carboxylesterase 1 (CES1), mobilizing free fatty acids (FFAs) from lipid droplets to fuel β-oxidation (FAO) during starvation. CES1 also prevents toxic lipid peroxidation, linking NF-κB to ferroptosis resistance [380]. In gastric cancer, fatty acid-induced hyper-O-GlcNAcylation activates NF-κB to upregulate CD36, a fatty acid transporter, promoting metastasis [381]. Studies in Drosophila revealed a role for NF-κB/Relish in regulating lipid metabolism during metabolic adaptation, which involves limiting the transcriptional function of Foxo [382]. A recent study highlighted an immunometabolic-NF-κB interplay in pancreatic cancer, in which immunotherapy-activated CD8^+^ T cells upregulated fatty acid synthesis (particularly oleic acid), thereby inducing lysosomal membrane permeabilization and cathepsin B release [383]. Cathepsin B, in turn, activates NF-κB to drive the expression of lipocalin-2, causing lipocalin-2-mediated recruitment of immunosuppressive PMN-MDSCs to the TME and antitumor resistance. GLP1-mediated suppression of oleic acid synthesis disrupts this immunosuppressive axis, illustrating how NF-κB couples immunometabolic rewiring in T cells to immune evasion [383]. These findings position NF-κB as a multifunctional rheostat for lipid flux, balancing energy provision with redox stability in cancer cells while simultaneously orchestrating immunometabolic programs that sustain immunosuppressive microenvironments.

In summary, NF-κB sits at the nexus of inflammation and metabolism and reprograms the glycolysis, OXPHOS, and lipid pathways to fuel tumorigenesis and immune escape (Fig. 4). Its context-dependent regulation—shaped by p53 status, nutrient availability, and cell type—highlights the complexity of targeting this pathway. Unraveling the NF-κB-metabolism axis may unlock novel strategies to disrupt the protumorigenic niche of the TME.

### NF-κB’s context-dependent dual roles in cancer

The NF-κB signaling pathway exhibits a striking duality in cancer biology, acting as both a tumor promoter and a tumor suppressor depending on the cellular context, tumor type, stage, and microenvironmental cues. Its dual functionality arises from the diversity of its subunits, the activation of distinct canonical (RelA/p50) or noncanonical (RelB/p52) pathways, and intricate crosstalk with other signaling networks. In many malignancies, NF-κB drives tumor progression by orchestrating inflammation, immune evasion, and survival pathways. In TAMs, canonical NF-κB (RelA/p50) promotes proinflammatory cytokine production (e.g., TNF-α, IL-6) in inflammation-driven cancers (e.g., colitis-associated colon cancer) or immunosuppressive IL-10 secretion in breast and ovarian cancers, fostering tumor growth and immune escape [174, 384–386]. Similarly, NF-κB activation in MDSCs enhances the levels of immunosuppressive mediators (IL-10 and IDO) in an NF-κB-dependent manner, which is correlated with poor prognosis [303, 387–389]. These protumorigenic roles are further amplified in the cancer cells themselves. Constitutive NF-κB activation in tumor cells increases the expression of prosurvival genes (e.g., BCL2, XIAP), angiogenic factors (VEGF, IL-8), and metastasis-promoting molecules (CXCR4, MMPs) [268, 390, 391]. In colitis-associated cancer, epithelial NF-κB suppresses the apoptosis of premalignant cells, whereas in EBV-associated lymphomas, viral proteins (e.g., LMP1) hijack NF-κB to sustain proliferation [174, 392–394], highlighting its context-dependent oncogenic versatility.

However, NF-κB also has tumor-suppressive functions, particularly in regulating antitumor immunity. In DCs, NF-κB enhances antigen presentation and IL-12 production, which are critical for antitumor CD8^+^ T-cell responses [93, 395, 396]. NK cell cytotoxicity against tumors relies on NF-κB-driven perforin and granzyme B production [307, 308]. Even within adaptive immunity, NF-κB exhibits duality: while promoting effector T-cell proliferation and cytokine production, it simultaneously maintains Treg fitness, balancing immune activation and tolerance [333, 397–401]. Hepatocellular carcinoma (HCC) further exemplifies this paradox. In hepatocellular carcinoma (HCC), hepatocyte-specific NF-κB activation inhibits chemically induced tumorigenesis by suppressing JNK-driven inflammation and necrosis [402–405]. Similarly, Nrf2-NF-κB crosstalk in liver epithelia maintains redox homeostasis, preventing malignant transformation [406]. The microenvironment emerges as a critical arbitrator of the dual roles of NF-kB. Hypoxia, stroma-derived TGF-β, and immune checkpoint signals such as PD-L1 reshape NF-κB activity across cellular compartments.

Spatial and temporal heterogeneity complicates therapeutic targeting, as systemic NF-κB inhibition may impair antitumor immunity while alleviating immunosuppression. Therapeutic strategies must therefore address this complexity. Inhibiting specific subunits such as c-Rel in MDSCs or Tregs could suppress protumor signaling while sparing antitumor DC or NK cell functions. Combinatorial approaches—pairing NF-κB inhibitors with immunotherapies—might exploit their immune-activating roles in DCs and T cells while counteracting their prosurvival effects in cancer cells. Advances in cell type-specific targeting and single-cell analyses will be pivotal in deciphering spatiotemporal NF-κB dynamics, enabling precision interventions tailored to tumor stage, subtype, and microenvironmental cues. Ultimately, understanding the context-dependent duality of NF-kB is not just a biological curiosity but a roadmap for reconciling its opposing roles in cancer therapy.

### NF-κB and therapeutic resistance

NF-κB activation is a hallmark of many cancers and is driven by chronic inflammation, oncogenic mutations, or therapy-induced stress. It promotes tumorigenesis, metastasis, and therapeutic resistance through multiple mechanisms. NF-κB upregulates antiapoptotic proteins (e.g., Bcl-2, Bcl-xL, XIAP, and cIAP1/2) that counteract chemotherapy- or radiation-induced apoptosis. For example, genotoxic therapies activate NF-κB via ataxia-telangiectasia mutated (ATM)-mediated IKK phosphorylation, enabling tumor cell survival despite DNA damage [407]. In addition to promoting apoptosis evasion, NF-κB promotes EMT and CSC maintenance by upregulating the expression of transcription factors such as Twist, ZEB1/2, and Slug, which repress E-cadherin and induce the expression of mesenchymal markers such as N-cadherin and vimentin. This EMT program enhances metastatic potential and chemoresistance [408]. In glioblastoma, hypoxia-induced NF-κB activation drives EMT and CSC enrichment via Snail and HIF-1α, fostering resistance to temozolomide [409].

NF-κB also contributes to therapy resistance by regulating drug efflux. It directly upregulates multidrug resistance (MDR) genes such as MDR1 (ABCB1), which encodes P-glycoprotein to efflux chemotherapeutics such as doxorubicin [410]. The TME further amplifies resistance through NF-κB-mediated crosstalk. CAF-derived IL-8 promotes chemoresistance in human gastric cancer via NF-κB activation [411]. In ovarian cancer, NF-κB-driven CCL2 and PI3K/Akt activation confer paclitaxel resistance by enhancing macrophage recruitment and survival signaling [412].

Overall, NF-κB is a master regulator of therapy resistance, acting through antiapoptotic signaling, EMT/CSC plasticity, and TME modulation. While current inhibitors face challenges in specificity, emerging strategies—precision targeting, combination therapies, and immune modulation—hold promise for overcoming resistance. Future research must address the dynamic interplay between NF-κB and other oncogenic pathways to develop context-specific therapies.

## NF-κB as a therapeutic target in inflammation and cancer

**Fig. 6:**
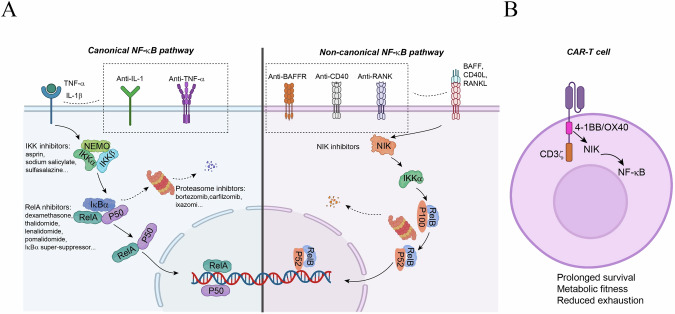
NF-κB-targeted therapeutics in inflammatory diseases and cancer. Abnormal activation of NF-κB is linked to inflammatory diseases and cancer, making this pathway an attractive therapeutic target. **A** Targeting the canonical and noncanonical NF-κB pathways with various small-molecule inhibitors or receptor-blocking monoclonal antibodies. **B** Incorporating the signaling domain of 4-1BB or OX40 into chimeric antigen receptors (CARs) in CAR-T-cell therapy promotes the activation of NF-κB, especially the NIK-dependent noncanonical NF-κB pathway, and improves the efficacy of cancer immunotherapy. The NIK pathway promotes the survival and metabolic fitness and reduces the exhaustion of CAR-T cells. The images were created with BioRender (www.biorender.com)

Given its critical involvement in inflammation and cancer, NF-κB is an attractive therapeutic target. Although very few specific NF-κB inhibitors are currently used in clinical practice, a variety of commonly employed anti-inflammatory or anticancer drugs modulate the NF-κB pathway as part of their mechanism of action. Some other NF-κB-targeting agents have been evaluated or are under evaluation in clinical trials (Table 1). In addition to small molecules, NF-κB-targeting agents also include monoclonal antibodies, noncoding RNAs and cellular therapy products (Fig. 6).

**Table 1:** NF-κB pathway inhibitors in clinic

Name	Mode of action	Clinical trial	Status	Indication	Efficacy	Adverse effects
SAR113945	IKKβ inhibitor	NCT01463488 Phase I	Completed	Osteoarthritis	No significant symptom relief	Nasopharyngitis, joint pain and swelling, and difficulty walking
		NCT01511549 Phase I	Completed	Osteoarthritis		
		NCT01113333 Phase I	Completed	Osteoarthritis		
		NCT01598415 Phase IIa	Completed	Osteoarthritis		
IMX-110	IKK & RelA inhibitor	NCT03382340 Phase 1/2a	Active	Advanced solid tumors	N/S	N/S
Withania somnifera		NCT05031351 Phase II	Unknown status	Amyotrophic lateral sclerosis (NIALS)	N/S	N/S
IMD-1041	IKKβ inhibitor	NCT00883584 Phase IIa	Unknown status	Chronic obstructive pulmonary disease (COPD)	N/S	N/S
MLN4924 (Pevonedistat)	IκBα degradation inhition	41 trials Phase I/II/III	21 trials completed	Tumor	Mixed results, some showing efficacy	Peripheral edema, pyrexia, diarrhea, pneumonia and fatigue
Curcumin (Turmeric extract)	IKK & RelA inhibitor	127 trials Phase I/II/III	71 trials completed	Osteoarthritis, depression, schizophrenia cognitive decline, solid tumors, and inflammatory diseases	Mixed results, some showing efficacy	Diarrhea, loose stools, headache, anxiety, and itching
Bortezomib (Velcade®)	IκBα degradation inhition	166 trials Approved or Phase/II/III	96 trials completed	Tumor	Mixed results, approved for MM	Heart failure, hypoxia and sepsis
Thalidomide/Lenalidomide	RelA inhibitor	81 trials Approved or Phase/II/III	39 trials completed	Tumor	Mixed results, approved for MM	Peripheral neuropathy, constipation and sedation
Disulfiram (Antabuse)	IκBα degradation inhition, and RelA inhibitor	76 trials Approved or Phase I/II	42 trials completed	Cocaine dependence, tumor, Post-treatment Lyme disease syndrome and HIV latency reversal	Mixed results approved for alcohol use disorder	Headache,nausea, drowsiness and rash

### IKK inhibitors

#### Aspirin and sodium salicylate

Aspirin (acetylsalicylic acid) and sodium salicylate are closely related compounds that are both derived from salicylic acid and classified as nonsteroidal anti-inflammatory drugs [413]. Research suggests that the anti-inflammatory properties of aspirin are partially attributed to its inhibition of IKKβ, which prevents the degradation of IκBα and thereby blocks the activation of NF-κB and the expression of genes involved in inflammatory responses [414, 415]. In addition to their anti-inflammatory effects, aspirin and sodium salicylate have potential antitumor activity and cancer-preventive effects, attracting great interest in their use for cancer treatment and prevention [416–419]. For example, aspirin has been shown to promote cell death in osteosarcoma cell lines and to suppress the migration, invasion, and metastasis of osteosarcoma both in vitro and in vivo. This tumor-suppressive effect involves inhibition of the NF-κB pathway, as evidenced by reduced nuclear localization of RelA and decreased expression of NF-κB target genes, including cIAP, XIAP, BCL2, and Survivin [420]. Furthermore, the hydrogen sulfide-releasing agent aspirin inhibits the proliferation and promotes the apoptosis of estrogen receptor-negative breast cancer cells, and the underlying mechanism involves the inhibition of IκBα phosphorylation, leading to reduced translocation of RelA into the nucleus [421]. Similarly, sodium salicylate induces a shift from a proliferative to an apoptotic phenotype in human leukemia cells by inhibiting the NF-κB response, decreasing FLIP levels, and restoring TNF-induced apoptosis [422].

#### Sulfasalazine

Sulfasalazine is a disease-modifying antirheumatic drug that has been widely used to treat IBD, RA, and other autoimmune conditions [423, 424]. Sulfasalazine is recognized as a classic inhibitor of NF-κB that acts by inhibiting the activity of IKK [425, 426]. Sulfasalazine ameliorates acute colitis in a mouse model induced by trinitrobenzene sulfonic acid, which involves the inhibition of NF-κB activation as well as the downregulation of NF-κB signaling components, such as TLR4, MyD88, and NF-κB RelA [427]. Moreover, sulfasalazine has been shown to alleviate LPS-induced acute lung injury by inhibiting the nuclear translocation of RelA [428]. Additionally, sulfasalazine has demonstrated potential in treating certain types of tumors, as it can promote apoptosis in U251 glioblastoma cells, an effect associated with the inhibition of NF-κB signaling [429].

#### IKKβ inhibitors

Many IKKβ inhibitors have demonstrated efficacy in various preclinical models of cancer and inflammatory diseases. For example, MLN-120B inhibits the growth of multiple myeloma (MM) cells in both cell lines and a clinically relevant severe combined immunodeficiency (SCID)-hu mouse model [430]. Additionally, MLN-120B reduces paw swelling in a dose-dependent manner and provides significant protection against arthritis-induced weight loss as well as cartilage and bone erosion in a rat model of RA [431]. BAY11-7821, another inhibitor of IKK, suppresses the proliferation and inflammation of glioma cells by inducing autophagy [432]. Additionally, IMD-0560 inhibits the phosphorylation of IκBα and the subsequent nuclear translocation of NF-κB. It reduces the production of inflammatory cytokines and chemokines, including IL-6, IL-8, and MCP-1, thereby protecting mice from collagen-induced arthritis [433]. IMD-1041, a prodrug of IMD-0354, is another IKKβ inhibitor that has been shown to alleviate NF-κB-mediated cardiac dysfunction, kidney injury, and COPD [434, 435]. Despite promising preclinical results for IKKβ inhibitors in these disease models, there is limited optimism that IKKβ inhibitors will soon see widespread clinical use, since IKKβ is ubiquitously expressed and plays a critical role in many physiological processes. Moreover, the complex, context- and tissue-specific functions of the IKK/NF-κB signaling pathway have made it difficult to predict the net effect and clinical outcome of systemic inhibition. Safety concerns currently pose a significant barrier to the development of IKK inhibitor-based drugs. Indeed, only a small number of phase I/II clinical trials involving IKK inhibitors have been conducted (Table 1). For example, although four clinical trials evaluated SAR113945 in the treatment of osteoarthritis, they failed to demonstrate meaningful symptom relief in patients [436, 437]. The severe side effects of IKK inhibitors may be beyond NF-κB inhibition, since IKK also possesses NF-κB-independent functions, including protection of cells from TNF-induced death [103, 212, 438]. Thus, the inhibition of downstream steps, such as IκBα degradation, NF-κB nuclear translocation or transactivation, has been explored as alternative approaches.

### Inhibitors targeting RelA function or nuclear translocation

#### Dexamethasone

Dexamethasone is a synthetic glucocorticoid with potent anti-inflammatory and immunosuppressive properties, and it inhibits NF-κB activation via different mechanisms. Dexamethasone inhibits RelA nuclear translocation through the induction of IκBα synthesis, and it interferes with the binding of RelA to the basal transcription machinery [439–441]. Dexamethasone is widely used to treat various inflammatory, autoimmune, and neoplastic conditions [442]. Interestingly, red blood cell-mediated delivery of dexamethasone selectively inhibits the NF-κB pathway and reduces TNF-α production in macrophages [443]. Moreover, dexamethasone has demonstrated efficacy in alleviating acute pancreatitis by inhibiting the NF-κB pathway [444].

#### Thalidomide, lenalidomide and pomalidomide

Thalidomide, lenalidomide, and pomalidomide are immunomodulatory drugs that have been proposed to inhibit NF-κB through interfering with the binding of RelA to open chromatin [445], although they may also inhibit IKK [446]. These compounds share a common structural framework and mechanisms of action, including inhibition of angiogenesis, immune modulation, and anti-inflammatory effects. However, they differ in potency, clinical indications, and side effect profiles [447, 448]. Thalidomide has been shown to alleviate skin inflammation through the suppression of NF-κB activation in keratinocytes [449]. Specifically, thalidomide significantly reduced erythema and decreased inflammatory cell infiltration in the dermis of LL37-induced rosacea-like mice. Its efficacy appears to be mediated through the inhibition of NF-κB RelA phosphorylation and nuclear translocation [449]. Additionally, thalidomide has promising therapeutic potential in tumor treatment, as shown by its ability to inhibit lung cancer cell invasion and metastasis, which involves the suppression of NF-κB-mediated ICAM-1 expression [450]. Lenalidomide received FDA approval in 2003 for the treatment of relapsed or refractory MM. It impairs the NF-κB signaling pathway in bone cells, resulting in the suppression of osteoclastogenesis, survival factors and bone-remodeling markers in MM [451]. Furthermore, lenalidomide has demonstrated potential in the treatment of diffuse large B-cell lymphoma, particularly by selectively inhibiting the proliferation of the activated B-cell (ABC) subtype of diffuse large B-cell lymphoma cells and delaying tumor progression in a human xenograft model. Its antitumor effects are linked to the downregulation of IRF4, leading to the suppression of B-cell receptor-dependent NF-κB activity [452]. Additionally, lenalidomide has been shown to mitigate postinflammatory pulmonary fibrosis by inhibiting NF-κB signaling [453]. Pomalidomide, a next-generation immunomodulatory drug, has demonstrated efficacy in the treatment of relapsed or refractory MM. In MM cells, which are highly dependent on transcriptional regulation, pomalidomide inhibits NF-κB activity and thereby suppresses COX-2 gene transcription, an action that contributes to its therapeutic efficacy [454].

#### IκBα superrepressor

The IκBα superrepressor is a genetically engineered IκBα protein that lacks IKK phosphorylation sites, thereby preventing its inducible degradation and increasing its ability to inhibit the nuclear translocation of NF-κB family members, especially RelA and p50. Emerging preclinical evidence highlights the extensive application of IκBα superrepressors delivered via exosome systems in various disease models, including RA, acute respiratory distress syndrome, alcohol-associated liver injury, sepsis-induced organ damage, kidney ischemia‒reperfusion injury, and amyotrophic lateral sclerosis (ALS) [455–460]. These findings suggest that the IκBα superrepressor holds significant potential as a future therapeutic intervention in clinical practice.

### Proteasome inhibitors

Proteasome inhibitors are a class of small-molecule compounds that block proteasome activity, thereby preventing IκBα degradation and NF-κB activation [461]. Among them, bortezomib, carfilzomib, and ixazomib have been approved by the FDA for the treatment of MM [461]. Beyond MM, bortezomib has demonstrated the ability to alleviate skin lesions in an imiquimod-induced psoriatic mouse model, in which it inhibits NLRP3 inflammasome activation and NF-κB signaling, reducing psoriatic inflammation [462]. Carfilzomib has been reported to induce growth arrest and apoptosis in mantle cell lymphoma cells by inhibiting NF-κB activation [463]. Additionally, both carfilzomib alone and in combination with pistachio hull extract significantly suppressed the growth of breast cancer cell lines, an effect linked to the inhibition of the NF-κB pathway [464]. Carfilzomib also enhances doxorubicin-induced cytotoxicity and apoptosis in breast cancer cells [465]. Ixazomib has shown clinical benefits in MM tumors characterized by increased noncanonical NF-κB pathway activity [466].

### Monoclonal antibodies and recombinant proteins

#### IL-1 inhibitors

IL-1α and IL-1β are proinflammatory cytokines that bind to a common receptor, the IL-1 receptor (IL-1R), to activate the NF-κB and MAPK signaling pathways, thereby amplifying inflammation [467]. Dysregulated IL-1 signaling is implicated in various autoimmune and chronic inflammatory diseases. Several inhibitors of IL-1, including the IL-1β blocking antibody canakinumab, the IL-1 trapping fusion protein rilonacept, and the recombinant IL-1R antagonist anakinra, have been approved for the treatment of multiple inflammatory and autoimmune diseases, such as RA [468], cryopyrin-associated periodic syndrome (CAPS) [469, 470], and children and adult Still’s disease [471]. In addition to its role in inflammatory diseases, IL-1 dysregulation has been linked to nearly all types of human malignancies [472]. The therapeutic potential of IL-1 inhibitors in cancer treatment has been actively explored [473]. However, the role of IL-1 in regulating cancer development appears to be complex [472]. Although IL-1β is widely known as a cancer-promoting cytokine, the IL-1β-mediated inflammatory response has also been shown to suppress metastatic colonization, and IL-1β blockade promotes metastasis, although it reduces primary tumor growth [474, 475]. In addition, major differences exist between the functions of IL-1α and IL-1β [472]. These findings provide an explanation for the unsatisfactory efficacy of IL-1 inhibitor therapies. For example, a recent clinical trial (NCT03631199) investigating the combination of an IL-1 inhibitor with a PD-1 inhibitor in lung cancer treatment demonstrated clinically meaningful symptom control but did not significantly prolong progression-free survival (PFS) or overall survival (OS) [476]. In addition to clinical trials, more preclinical studies are warranted to better understand the functions of IL-1α and IL-1β in regulating different stages of cancer development.

#### Anti-TNF-α

To date, five anti-TNF-α biologic agents have received clinical approval: infliximab (Remicade®), adalimumab (Humira®), golimumab (Simponi®), certolizumab pegol (Cimzia®), and etanercept (Enbrel®). These biologics are extensively utilized in the management of autoimmune and chronic inflammatory disorders, including RA, psoriasis, IBD, and ankylosing spondylitis [477]. Recent advances in preclinical research have highlighted the therapeutic potential of novel anti-TNF-α peptide-based therapies. For example, a synthetic TNF-α blocking peptide has been shown to effectively suppress the NF-κB and MAPK signaling pathways, thereby attenuating inflammatory responses [478]. Additionally, another engineered peptide exhibited potent inhibition of NF-κB and MAPK activation in a murine model, significantly alleviating DSS-induced colitis [479]. These findings underscore the therapeutic potential of next-generation peptide inhibitors as potential treatments for inflammatory diseases.

#### Noncoding RNAs

Noncoding RNAs (ncRNAs) include primarily microRNAs (miRNAs), long noncoding RNAs (lncRNAs), and circular RNAs (circRNAs). Accumulating evidence suggests that ncRNAs contribute to the aberrant regulation of NF-κB signaling in tumorigenesis [480, 481]. Notably, NF-κB signaling may be inhibited or activated by miRNAs across various tumor models. This miRNA/NF-κB regulatory axis plays a critical role in modulating tumor proliferation, metastasis, and therapeutic responses to chemotherapy [480, 482–484]. LncRNAs and circRNAs are also capable of regulating NF-κB expression across different cancer types. Notably, their effects on NF-κB signaling are generally indirect and are primarily mediated through the modulation of miRNAs [485, 486]. These findings highlight the potential of targeting ncRNAs to modulate NF-κB activity as a promising strategy for suppressing tumorigenesis driven by dysregulated NF-κB signaling. Indeed, anti-miRNA oligonucleotides have demonstrated effectiveness in inhibiting NF-κB activity in cancer [487].

### Adoptive T-cell therapy

Adoptive T-cell therapy (ACT) is a form of immunotherapy that involves reinfusing genetically modified or expanded T cells into patients to enhance the immune response against cancer or autoimmune diseases. It is one of the most promising approaches in cancer immunotherapy, particularly for hematologic malignancies and certain solid tumors. ACTs include tumor-infiltrating lymphocyte (TIL) therapy, CAR-T-cell therapy and TCR-T-cell therapy [488]. The basic structure of a CAR consists of an extracellular antigen-binding domain, a transmembrane domain, and an intracellular signaling domain, which usually includes costimulatory regulators such as 4-1BB, OX40, or CD28. Among them, 4-1BB CAR-T cells have demonstrated superior efficacy to CD28 CAR-T cells in both preclinical studies and clinical trials [489]. Mechanistically, 4-1BB can increase T-cell expansion and prolong persistence [490], which has been validated by several recent studies. Human CARs with a 4-1BB domain induce strong NF-κB activation through the recruitment of TRAF molecules, highlighting the crucial role of the TRAF-NF-κB axis in sustaining CAR-T-cell persistence following antigen stimulation [491]. Furthermore, compared with CD28 CAR-T cells, 4-1BB CAR-T cells exhibit greater ex vivo survival and subsequent expansion, which correlates with activation of the noncanonical NF-κB pathway. Inhibition of NIK by overexpression of a dominant-negative NIK peptide reduces 4-1BB CAR-T-cell expansion and survival, with the latter being attributed to noncanonical NF-κB-mediated suppression of the proapoptotic protein Bim [492]. Similarly, another TNF-receptor superfamily member, OX40, has been shown to increase CAR-T-cell persistence under repeated antigen stimulation [493]. This phenotype was likewise observed in TCR complex-based CAR-T cells (STAR-T cells) [494]. OX40 signaling reduces CAR-T-cell apoptosis by upregulating genes encoding Bcl-2 family members and enhances proliferation through increased activation of the NF-κB, MAPK, and PI3K-AKT pathways [495]. Consistent with these findings, ectopic expression of NIK, a kinase specifically targeted by TNFRs such as 4-1BB and OX40, prevents CD8^+^ T-cell exhaustion and promotes CD8^+^ T-cell metabolism and antitumor immunity in MC38 colon cancer and B16 melanoma mouse tumor models [110]. Taken together, these results suggest that activating the NF-κB pathway may increase the efficacy of ACT in tumors.

### Side effects and challenges

While NF-κB-targeted therapies are promising for cancer and autoimmune inflammatory diseases, their side effects are likely to resemble those of other immunomodulatory and anti-inflammatory agents. The most significant concern is the potential for compromised immune responses and increased susceptibility to infections due to the systemic and indiscriminate blockade of NF-κB signaling. As mentioned earlier, NF-κB plays a crucial role in acute inflammation by promoting beneficial responses to tissue damage and pathogen infection. Furthermore, NF-κB is involved in numerous homeostatic and developmental pathways. Therefore, systemic blockade of NF-κB signaling is inevitably linked to weakened immune function, increasing vulnerability to infections. Moreover, in the context of cancer treatment, NF-κB inhibition-induced immunosuppression may also weaken antitumor immunity, influencing therapeutic efficacy. Thus, striking a delicate balance between suppressing the pathological functions of NF-κB and preserving its normal cellular functions is critical. For example, targeting NF-κB signaling in a tissue- or cell-specific manner may improve therapeutic efficacy and reduce systemic toxicity, as attempted in some mouse model studies [496, 497]. In this context, the use of nanoparticles to selectively target cancer cells with NF-κB inhibitors represents a promising precision targeting strategy [498].

Another concern involves indiscriminate apoptosis caused by NF-κB inhibition in both target and nontarget cells. While increased apoptosis is a mechanism of action for NF-κB inhibitors in various tumors, it may not be beneficial in treating autoimmune and inflammatory diseases. For most inflammatory diseases, the ideal outcome is to selectively inhibit the production of proinflammatory factors by immune cells without jeopardizing their survival and functions in other aspects of immune responses. More importantly, normal epithelial [499] and cardiac cells [500] may also undergo apoptosis in response to NF-κB modulation, raising concerns about potential toxicity.

Finally, NF-κB inhibition may cause neutrophil abnormalities. As mentioned above, prolonged NF-κB inhibition has been shown to promote neutrophil survival, leading to increased neutrophilia and neutrophil-mediated autoinflammation due to aberrant IL-1β secretion. Taken together, to improve the safety profile of NF-κB inhibitors, close monitoring for infections, inflammatory rebound, and organ-specific toxicity is essential. Combining NF-κB inhibitors with other therapies may amplify these risks, necessitating careful dosing and patient selection strategies. Clinical trials will be critical in defining the precise safety profile of these agents.

## Concluding remarks

The transcription factor NF-κB functions as a master regulator of immune responses, inflammation, and oncogenesis, acting as a double-edged sword in both health and disease. In acute inflammation, NF-κB activation serves as a protective mechanism by promoting rapid immune cell recruitment and facilitating pathogen clearance. However, in the context of chronic inflammatory and autoimmune diseases, its dysregulation drives sustained proinflammatory cytokine production, persistent immune cell infiltration, and increased oxidative stress, thereby contributing to tissue damage. In cancer, persistent activation of NF-κB supports cancer cell proliferation and survival, angiogenesis, metastasis, and therapy resistance; however, physiological NF-κB function is required for anticancer immunity. This dual-function nature highlights the complexity and challenge of targeting NF-κB as a therapeutic strategy.

The immense complexity of the NF-κB pathway presents both an advantage and a challenge in the development of NF-κB-targeted therapies. The large number of proteins involved in the NF-κB signaling cascade provides multiple levels of regulatory control, allowing for fine-tuned therapeutic intervention. However, to increase the potency and safety of NF-κB-based therapies, future research should focus on three key principles: cell type- or tissue-specific targeting. Targeting NF-κB signaling in specific cell types or tissues can help minimize side effects and preserve normal immune function. This can be achieved through antibody‒drug conjugates (ADCs) and nanoparticle-based delivery systems. ADCs can couple monoclonal antibodies or small molecules with tumor cell- or immune cell-specific ligands to selectively target cancer cells or immune cells. NPs can deliver NF-κB inhibitors directly to tumors or inflamed tissues, thereby reducing systemic toxicity. Second, there are combinatorial approaches. We can either combine low doses of NF-κB inhibitors with other targeted therapies to reduce toxicity while maintaining efficacy or pair NF-κB inhibitors with immunotherapy, chemotherapy, or kinase inhibitors to overcome resistance. The third factor is patient evaluation and selection. Identifying patient subgroups most likely to benefit from NF-κB pathway inhibition can maximize therapeutic success. By revealing the spatial, temporal, and disease-specific dynamics of NF-κB signaling, it may be possible to harness its therapeutic potential while minimizing risks. A deeper understanding of NF-κB regulation will pave the way for the development of safer and more effective treatments for inflammation and cancer.
